# Assessing the impact of waterfront trail aesthetics on psychological restoration in urban environments: a deep learning and random forest approach

**DOI:** 10.3389/fpubh.2025.1757145

**Published:** 2026-01-30

**Authors:** Yongshu Wu, Yuhan Wang, Juan Wang, Junyi Li, Yinghong Ding, Yaqin Ding, Yuxuan Wu, Zhipeng Zhu, Xiaoling Xu

**Affiliations:** 1 College of Landscape Architecture and Art, Fujian Agriculture and Forestry University, Fuzhou, China; 2 College of Architecture and Planning, Fujian University of Technology, Fuzhou, China

**Keywords:** aesthetic preference, psychological restoration, random Forest analysis, semantic segmentation, waterfront trails

## Abstract

Urban waterfront trails can promote psychological restoration, yet how specific landscape attributes shape scenic beauty and multidimensional restorative perception across different revetment types remains unclear. This study quantified 15 landscape variables (F1–F15) across 30 urban waterfront trail sites and examined their associations with scenic beauty estimation (SBE) and four restorative dimensions (emotional, cognitive, physiological, and behavioral). Spearman correlation analysis, non-parametric group comparisons, and Random Forest modeling were applied to identify key predictors, with variable importance interpreted as predictive contributions rather than causal mechanisms. Partial-mediation structural equation models (SEM) were then constructed for the overall sample and for artificial, mixed, and natural revetment types. Results indicated that F4 was the most influential predictor of SBE, followed by F7, F13, F2, and F5. SEM analyses showed that SBE functioned as a central mediator linking landscape attributes to restorative outcomes: F4 was positively associated with SBE (*β* = 0.460), and SBE positively predicted all four restorative dimensions (*β* = 0.802–0.917), with additional direct paths indicating partial mediation. Grouped SEMs further revealed revetment-type-specific pathway structures, suggesting that revetment context conditions which landscape elements most strongly relate to perceived beauty and psychological restoration.

## 1 Introduction

The rapidly advancing process of urbanization has brought considerable convenience while profoundly transforming people’s living environments and lifestyles. Dense building development, congested traffic, fast-paced daily life, and significantly reduced contact with nature have collectively contributed to increased psychological pressure among urban residents. Sensory overload, social alienation, and mental health problems have become increasingly prominent (1–3). Studies have shown that insufficient contact with nature is an important environmental factor contributing to mental health disorders such as stress, anxiety, and even depression (4, 5). In this context, identifying and optimizing urban spaces that can effectively support psychological restoration has become an urgent and important issue in the fields of urban planning and landscape design.

Urban waterfront spaces, as natural–artificial composite environments embedded within cities, have attracted widespread attention due to their potential physical and psychological restorative benefits. Numerous studies have confirmed that walking in urban natural environments can effectively alleviate stress among young people (6, 7). Moreover, compared with single green spaces, environments that incorporate water bodies generally exhibit stronger restorative effects and higher aesthetic appeal (8). As a key component of urban waterfront areas, waterfront promenades play an important role in enhancing residents’ mental health and social well-being.

Despite the growing body of research on waterfront environments, existing literature has largely focused on macro-level planning, ecological functions, and overall landscape structure, while systematic studies treating waterfront promenades as independent, experience-oriented landscape elements remain relatively limited. Early research primarily emphasized spatial form and ecological configuration, whereas more recent studies have gradually incorporated perspectives such as aesthetic evaluation and restorative effects (9, 10). However, the influencing factors of aesthetic preference and restorative potential in waterfront promenades are often examined in isolation, and systematic, quantitative studies that integrate both dimensions are still lacking.

From a methodological perspective, the Scenic Beauty Estimation (SBE) method is one of the most widely used visual evaluation systems. Developed by Daniel et al. (11), SBE quantifies landscape aesthetic preferences through participatory public assessments and has been extensively applied in visual landscape evaluation. Restorative assessment, in contrast, focuses on a landscape’s capacity to alleviate psychological stress and restore attention. Research suggests that in certain environments, human attention can be involuntarily drawn, thereby interrupting negative thoughts, suppressing negative emotions, and eliciting positive emotions, ultimately restoring diminished cognitive and behavioral capacities (12). Most existing studies on restorative effects are based on Kaplan’s Attention Restoration Theory (ART) and Ulrich’s Stress Reduction Theory (SRT) [KAPLAN; (13)]. According to ART, prolonged and highly focused attention leads to attention fatigue, which can be alleviated through interaction with natural environments. Furthermore, being away, extent, fascination, and compatibility are identified as four key characteristics that determine whether an environment has restorative potential [KAPLAN; (13)]. SRT emphasizes that positive emotions experienced in an environment are critical for stress reduction (14).

The aesthetic preference and restorative evaluation of waterfront promenade landscapes are influenced by multiple factors. For example, appropriate proportions of green and blue spaces can provide comfortable and pleasant visual experiences, while plant growth conditions and vertical layering affect landscape richness and aesthetic quality (3, 15). In addition, factors such as commercial facilities and visual complexity may negatively affect aesthetic and restorative quality. Excessive commercial facilities can undermine the naturalness and harmony of the landscape, while overly high visual complexity may lead to feelings of confusion and discomfort. Although ART and SRT provide important conceptual foundations for understanding restorative environments, the objective quantification of visual landscape elements in empirical studies remains relatively insufficient. Consequently, the internal mechanisms through which different visual landscape elements jointly influence aesthetic experience and perceived restoration have not yet been fully elucidated.

In recent years, advances in computer vision and machine learning have provided new opportunities to overcome these methodological limitations. Image-based semantic segmentation techniques enable the identification and quantification of landscape elements at the pixel level, decomposing complex visual scenes into measurable components with high accuracy in scene interpretation (16, 17). Compared with traditional methods, semantic segmentation can rapidly and accurately identify various landscape elements—such as vegetation, water bodies, sky, and buildings—and quantitatively analyze them. Its advantages include high efficiency, accuracy, and strong capability in handling complex scenes, thereby offering more objective data support for aesthetic preference and restorative evaluation. Random forest (RF) is an ensemble learning method primarily used for classification and regression tasks (18). By constructing multiple decision trees and aggregating their results, RF effectively reduces the risk of overfitting through dual randomness—random sampling of data and random selection of features—and can handle high-dimensional data and complex relationships, achieving high predictive accuracy (19–22). However, as a machine learning approach, random forest has been relatively underutilized in landscape evaluation studies. Applying this method can facilitate a deeper interpretation of evaluation results for waterfront promenade landscapes.

In summary, although existing studies on urban waterfront promenades have made some progress in aesthetic preference and restorative evaluation, several limitations remain. On the one hand, research on factors influencing aesthetic preference and restorative potential is neither comprehensive nor sufficiently in-depth, lacking systematic analysis and quantitative approaches. On the other hand, traditional on-site evaluation methods are often constrained by abstract quantification or reliance on qualitative analysis, resulting in limited data accuracy and precision. In contrast, using landscape images as evaluation objects is not only more efficient but also effectively reduces confounding factors. Against this background, this study takes waterfront promenades in Fuzhou, China, as the research object to systematically investigate visitors’ aesthetic preferences and perceived restorative potential. Specifically, this study: (1) employs image semantic segmentation techniques to objectively quantify visual landscape elements along waterfront promenades; (2) combines on-site questionnaire surveys to obtain visitors’ subjective evaluations of landscape aesthetics and restorative experiences; and (3) constructs random forest models to analyze the relationships between quantified landscape elements and perceived responses, supplemented by structural equation modeling to further examine the direct and indirect associations among key variables. By integrating image-based objective analysis with perception-based subjective evaluation, this study aims to more comprehensively and methodologically robustly reveal the mechanisms through which waterfront promenade landscapes influence aesthetic experience and psychological restoration, thereby providing scientific evidence for the design and optimization of restorative urban waterfront spaces.

## 2 Materials and methods

### 2.1 Study area

Fuzhou is located in the eastern coastal area of Fujian Province, downstream of the Min River, bordering the Taiwan Strait to the east. Characterized by a typical subtropical monsoon climate, it experiences warm, humid conditions and abundant rainfall, which provide a stable water supply for the urban river system (1). The topography is predominantly composed of interlacing hills and plains. The Min River flows through the city, and the urban area features a dense network of over 30 internal rivers—including the Jin’an River, Baima River, Guangming Gang, and Antai River—forming an extensive urban water system with diverse waterfront space types. By 2021, the city had established more than 500 such Beaded Parks, 270 of which are waterfront-type parks. This study focuses on the waterfront trails within Fuzhou’s Beaded Parks to explore their landscape characteristics and functional performance.

**Figure 1:**
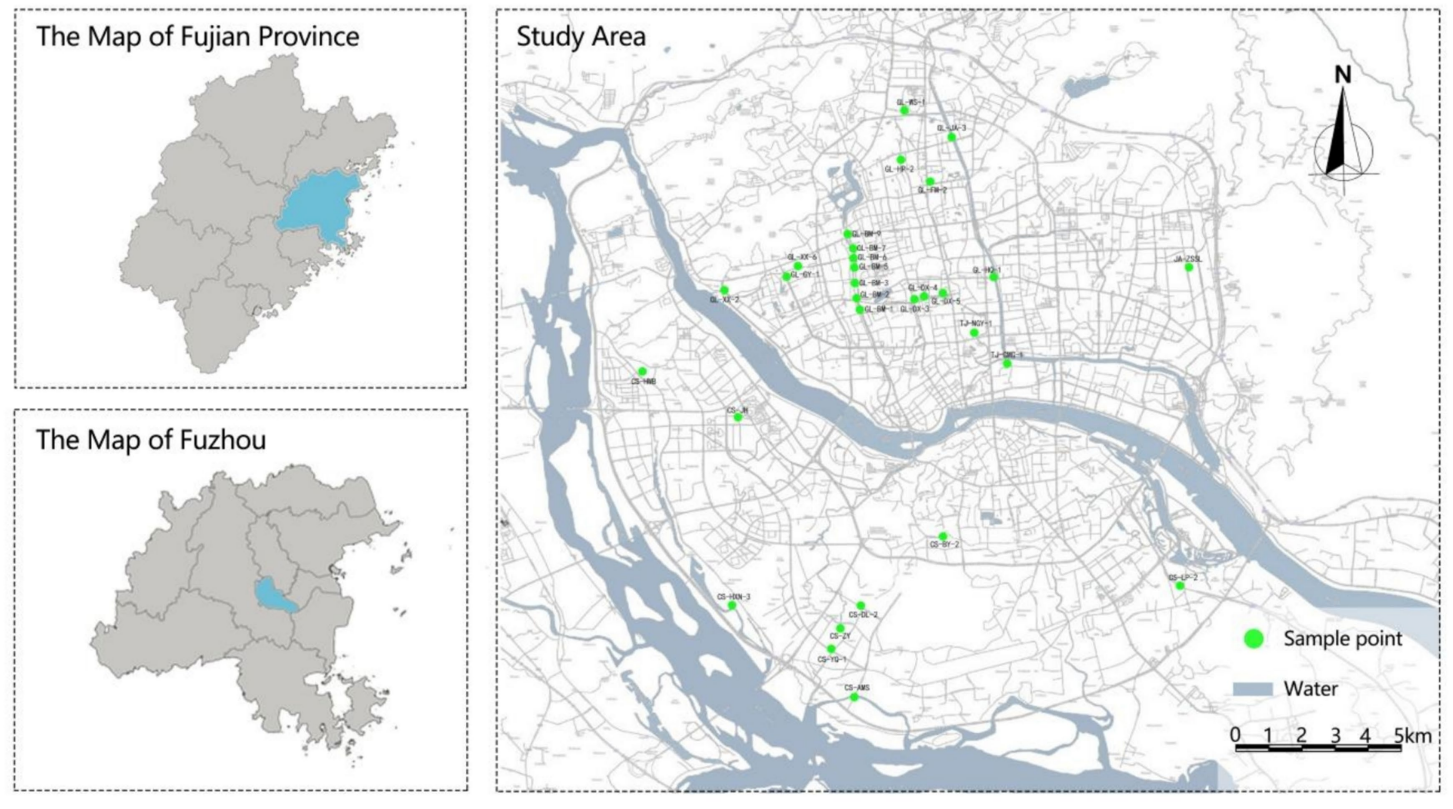
Distribution map of survey plots.

Within the waterfront trail system of Fuzhou, the study sites were categorized into three main types based on the construction form of the river revetment: Artificial Revetment (A), Natural Revetment (N), and Mixed Revetment (M). A total of 120 sample sites were investigated (Figure 1). Site selection was required to meet the following criteria: (1) the river must be directly visible from the trail space, and preliminary surveys confirmed consistent pedestrian flow; (2) the greenbelt must include vegetation structures such as tree-shrub-herb, tree-herb, or shrub-herb layers; (3) the river width must cover three categories: ≤ 2 m (narrow), 2–4 m (medium), and > 4 m (wide). The sample sites were coded using the format “Area Abbreviation-Road Number-Sequence Number” (e.g., Gulou District: GL-XX-1, Cangshan District: CS-XX-1, Jin’an District: JA-XX-1, Taijiang District: TJ-XX-1) to ensure precise spatial positioning.

### 2.2 Data collection and methodology

#### 2.2.1 Waterfront trail landscape image collection

This study employed a standardized photographic method to collect urban waterfront trail landscape data. The image collection was conducted between June 30 to July 5, 2023, from 7:00 a.m. to 11:00 a.m. A Sony camera, fixed on a tripod at a height of 1.6 meters above ground to simulate the human eye-level perspective, was used for systematic photography (23). Strict shooting protocols were enforced to ensure data quality: operations were carried out under clear weather conditions, maintaining a frontal lighting angle; all images were required to fully capture the water body and waterfront space, while avoiding interference from non-landscape elements such as pedestrians and vehicles. A preliminary set of 234 landscape images was acquired, with an output resolution of 1,250 × 600 pixels. After a rigorous screening process that excluded duplicate scenes, images with abnormal lighting, and those of overly similar types, cluster analysis was applied to ultimately identify 30 representative waterfront trail landscape images as the final study samples, as shown in Table 1.

**Table 1:** Classification of spatial types and site selection of waterfront trails in Fuzhou.

Number	Region	Sample point	Structural type	Number	Region	Sample point	Structural type
1	Jin’an District	JA-ZSSL	Artificial Revetment	16	Gulou District	GL-BM-5	Mixed Revetment
2	Taijiang District	TJ-GMG-1	Natural Revetment	17		GL-BM-6	Natural Revetment
3		TJ-NGY-1	Mixed Revetment	18		GL-BM-7	Natural Revetment
4	Cangshan District	CS-AMS	Natural Revetment	19		GL-BM-9	Artificial Revetment
5		CS-BY-2	Artificial Revetment	20		GL-DX-3	Mixed Revetment
6		CS-DL-2	Artificial Revetment	21		GL-DX-4	Artificial Revetment
7		CS-JH	Artificial Revetment	22		GL-DX-5	Artificial Revetment
8		CS-HXN-3	Mixed Revetment	23		GL-FM-2	Artificial Revetment
9		CS-LP-2	Artificial Revetment	24		GL-GY-1	Artificial Revetment
10		CS-HWB	Mixed Revetment	25		GL-HP-2	Artificial Revetment
11		CS-YQ-1	Natural Revetment	26		GL-HQ-1	Artificial Revetment
12		CS-ZY	Mixed Revetment	27		GL-JA-3	Mixed Revetment
13	Gulou District	GL-BM-1	Natural Revetment	28		GL-WS-1	Artificial Revetment
14		GL-BM-2	Natural Revetment	29		GL-XX-2	Mixed Revetment
15		GL-BM-3	Natural Revetment	30		GL-XX-6	Artificial Revetment

#### 2.2.2 Aesthetic preference assessment

The Scenic Beauty Estimation (SBE) method, established by Daniel et al. (11), is a widely used psycho-physical landscape assessment approach in landscape architecture. Objective scenic beauty metrics are derived by applying data standardization to eliminate individual judgment scale differences. Its core lies in linking subjective perception with the objective attributes of the landscape, and it utilizes Random Forest models to identify key factors influencing aesthetic quality. The questionnaire for aesthetic preference assessment employed a Likert scale format, establishes a measurement level for research by using standardized response options in surveys. It presents a series of statements, and respondents indicate their level of agreement using typically 7-point options ranging from “Strongly Agree” to “Strongly Disagree” (Equation 1).

$$Z_{\mathrm{ij}}=(R_{\mathrm{ij}}-\bar{R_{j}})/S_{j}$$ (1)

In the formula, $Z_{\mathrm{ij}}$ represents the standardized value of the j-th evaluator’s rating for the i-th waterfront trail landscape’s scenic beauty; $R_{\mathrm{ij}}$ denotes the raw score given by the j-th evaluator to the i-th waterfront trail landscape; $\bar{R_{j}}$ is the mean score given by the j-th evaluator across all waterfront trail landscape; and $S_{j}$ signifies the standard deviation of the scores given by the j-th evaluator for all landscapes (11).

Evaluations of landscape aesthetic value by different social groups show significant statistical convergence. The 30 selected representative samples were categorized based on revetment type into Natural Revetment, Artificial Revetment, and Mixed Revetment. These three categories of samples were arranged in a random order to create the survey questionnaire. The questionnaire used a 7-point Likert scale (where 1 represented “Very Dissatisfied” and 7 represented “Very Satisfied,” with points 2 through 6 representing intermediate levels). Participants were asked to rate the scenes based on landscape quality evaluation criteria.

#### 2.2.3 Restorative assessment

For the restorative assessment, this study employed a modified version of the Stress Recovery Rating Scale (SRRS) for the questionnaire survey. This scale was originally developed by Taiwanese scholar Han Ke-Zong based on the Attention Restoration Theory (ART) and the Stress Reduction Theory (SRT). It assesses the restorative impact of the environment on users across four dimensions: physiology, behavior, emotion, and cognition through a total of 17 items (24). Considering the characteristics of urban waterfront spaces, this study selected eight representative items from the original scale’s four dimensions to constitute the questionnaire. The questionnaire design consisted of two main parts (Table 2): The first part collected respondents’ socio-demographic characteristics. The second part required participants, after viewing the waterfront landscape images, to similarly use a 7-point Likert scale (1 = Strongly Disagree, 7 = Strongly Agree) to evaluate the restorative perceived effects of each scene. Data collection was carried out between January and February 2024. After field researchers provided detailed explanations of the questionnaire content, a total of 202 questionnaires were collected. Following screening, 12 invalid questionnaires were discarded, retaining 190 valid questionnaires for subsequent analysis.

**Table 2:** SRRS scale dimensions and items.

Dimensions	Item	Serial number
Emotion	How would you describe the impact of scenery on your emotional changes in the following environment?	
	Depressed → Happy	A1
	Anxious → Relaxing	A2
	Exhausted → Full of vitality	A3
Physiology	How would you describe the physiological response caused by the scenery in the following environment?	
	My breathing is accelerating.	B1
Cognition	How would you describe the impact of landscape on your cognition in the following environment?	
	I am very interested in the current environment.	C1
	My mental exhaustion is decreasing.	C2
Behavior	Which behavior would you identify with in the following environment?	
	I want to explore this place more deeply.	D1
	I want to visit here more often.	D2

### 2.3 Data processing and analysis

#### 2.3.1 Construction of the quantitative system

Based on field surveys of the Fuzhou waterfront landscape and consultations with relevant experts, environmental characteristics were decomposed into 15 pre-selected landscape evaluation factors across three aspects: spatial elements, natural elements, and artificial elements. The spatial elements include: sky openness, spatial enclosure, visual complexity, and color richness. The artificial elements include: building elements, pavement elements, pavement pattern, revetment type, facility elements, small structural elements, and waterfront enclosure degree. The natural elements include: blue view rate, green view rate, vegetation layers, and soil exposure degree. The aforementioned evaluation factors were sequentially numbered F1 through F15 (Table 3).

**Table 3:** Landscape element decomposition table of urban waterfront trail.

Type	Landscape elements	Computational method	Quantitative method	Serial number
Spatial elements	Sky openness	The proportion of the sky in the landscape view	Image semantic segmentation	F1
	Spatial enclosure	The proportion of vertical elements in the landscape view	Image semantic segmentation	F2
	Visual complexity	Complex index of landscape space composition	Matlab	F3
	Color richness	Color richness index of landscape spatial elements	Matlab	F4
Artificial elements	Building elements	The proportion of urban buildings in the landscape view	Image semantic segmentation	F5
	Pavement elements	The proportion of hard pavement in the landscape view	Image semantic segmentation	F6
	Pavement pattern	Cement = 1, Asphalt = 2, Bricks and Stones = 3, Wood = 4	Subjective assignment	F7
	Revetment type	Artificial = 1, Natural = 2, Artificial + Natural = 3	Subjective assignment	F8
	Facility elements	The proportion of landscape service facilities in the landscape view	Image semantic segmentation	F9
	Small structural elements	The proportion of decorative small structures in the landscape view	Image semantic segmentation	F10
	Waterfront enclosure degree	Degree of water boundary enclosure	Image semantic segmentation	F11
Natural elements	Blue view rate	The proportion of water bodies in the landscape view	Image semantic segmentation	F12
	Green view rate	The proportion of vegetation in the landscape view	Image semantic segmentation	F13
	Vegetation layers	The proportion of plants of different heights in the landscape view	Image semantic segmentation	F14
	Soil exposure degree	The proportion of bare soil in the field of view	Image semantic segmentation	F15

#### 2.3.2 Image semantic segmentation

DeepLabv3 is a semantic segmentation framework based on deep convolutional neural networks. Its advantage lies in using atrous convolution to replace traditional down-sampling operations, significantly reducing the loss of spatial information in feature maps while expanding the receptive field to capture long-range context. Its core architecture designs an Atrous Spatial Pyramid Pooling (ASPP) mechanism, which parallelly integrates local fine-grained features (dense sampling convolution), multi-scale contextual semantics (variable-rate atrous convolutions), and image-level statistical representations (global average pooling followed by spatial reconstruction modules). Combined with a strategy for progressively expanding the receptive field in the deeper layers of the backbone network, it achieves controllable context modeling (25, 26) It directly generates high-resolution segmentation results in an end-to-end manner, achieving a mean Intersection over Union (mIoU) of 79.4% and a total pixel accuracy (pixAcc) of 96.4% in tests, significantly improving the boundary recognition accuracy and semantic consistency for multi-scale objects in complex scenes (27).

**Figure 2:**
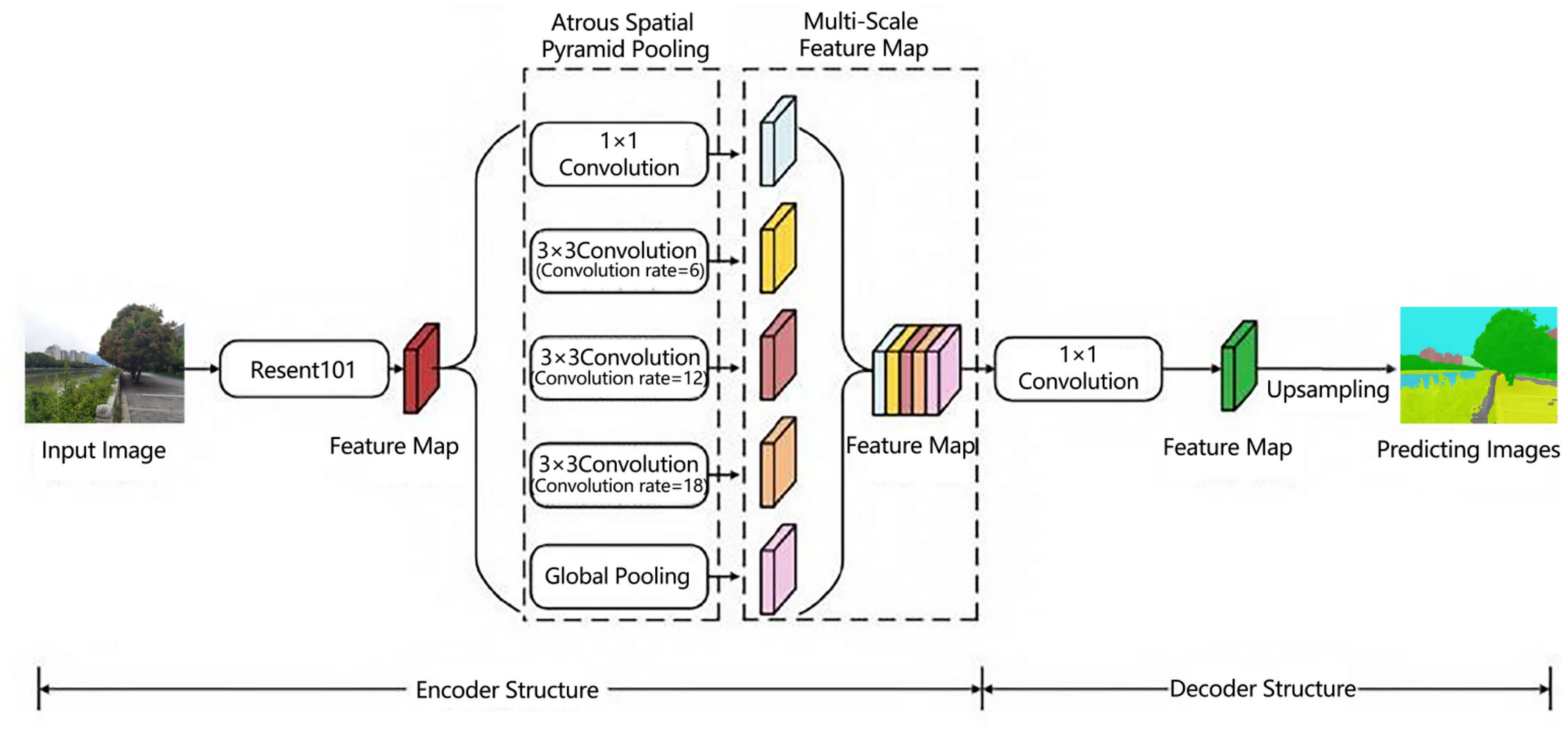
Deeplabv3 network structure (27).

In this study, the Cityscapes dataset was utilized to identify and segment common elements in the waterfront landscape. The waterfront trail landscape labels were categorized into 11 classes: building, sky, road, tree, medium/low layer vegetation, water, small structural, service facility, railing, soil, and vertical element (Figure 2).

#### 2.3.3 Spearman correlation, random forest model, and structural equation modeling

The 15 quantified landscape elements were treated as independent variables (X), while the SBE values and restorative indicators of the waterfront trail landscapes were, respectively, treated as dependent variables (Y). The Spearman correlation coefficient was calculated using Python to further verify the correlations between them. The Spearman rank correlation coefficient method is a non-parametric test used to measure the strength and direction of the relationship between variables (28). When data does not meet the assumption of normal distribution or contains outliers, Spearman correlation analysis is a preferable choice. The calculation formula for the Spearman correlation coefficient (ρ) is shown in Equation 2 (29).

$$\rho =1-\frac{6\sum d_{i}^{2}}{n(n^{2}-1)}$$ (2)

In the formula, d represents the difference between the ranks of variables X and Y, and n denotes the total number of samples. The value of ρ ranges from −1 to 1, where ρ = 1 indicates a perfect positive correlation with complete agreement in the ranking orders of the two variables; ρ = − 1 signifies a perfect negative correlation where one variable’s ranking is exactly opposite to the other’s; and ρ = 0 suggests no correlation exists between them.

The Random Forest model, introduced by Breiman (30), is a nonlinear and non-parametric ensemble algorithm based on regression trees. This model has been widely applied across numerous research fields (31, 32), as it offers higher predictive accuracy compared to traditional regression methods and can effectively assess the importance and relative contribution of independent variables. In this study, the four dimensions of restorative indicators (BD, CD, ED, and PD) and Scenic Beauty Estimation (SBE) values were used as dependent variables, while 15 quantified landscape elements were selected as independent variables. A Random Forest regression model was employed to analyze the impact of each independent variable on the restorativeness and scenic beauty of waterfront trails.

It should be noted that feature importance derived from the Random Forest model reflects the relative contribution of variables to predictive performance, rather than their causal influence on the response variable or evidence of underlying mechanisms, and therefore cannot be directly used for mechanistic inference. Based on this consideration, Structural Equation Modeling (SEM) was introduced as a complementary analytical approach, in which relationships among variables were explicitly specified under the guidance of theory and prior knowledge to examine the direct and indirect associations among key factors.

**Figure 3:**
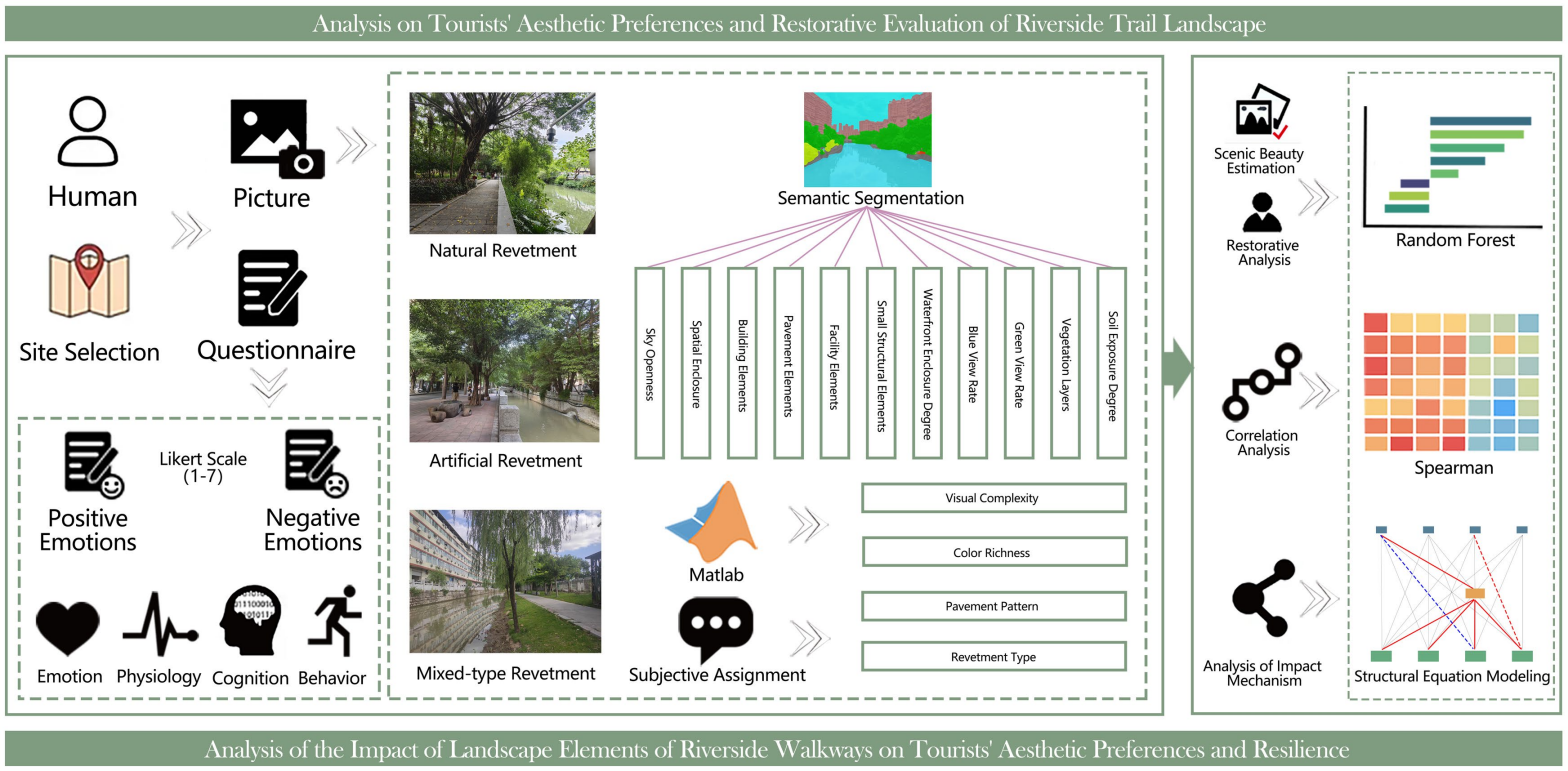
Research flow chart.

SEM was originally conceptualized by Jöreskog (33) and enables the simultaneous analysis of relationships between latent and observed variables within a unified framework. Its principal strength lies in the explicit specification of theoretically grounded structural pathways, allowing for the concurrent estimation of direct and indirect effects as well as overall model fit (34). SEM has therefore been widely applied in the social sciences, environmental behavior research, and landscape and planning studies. Review-based studies further indicate that SEM is particularly valuable in landscape performance and environmental perception research, as it facilitates the integration of subjective perceptual evaluations with objective environmental indicators, thereby supporting systematic analyses of multifactor interactions in complex environmental systems (Figure 3) is the overall flowchart of this experiment (35).

## 3 Results

Overall results showed a consistent typology gradient across outcomes: natural-type trails generally exhibited the highest levels, mixed-type trails were intermediate, and artificial-type trails were the lowest.

### 3.1 Analysis of aesthetic preference and restorative evaluation of different waterfront trail types

#### 3.1.1 Assessing aesthetic preferences across different waterfront trail types

**Figure 4:**
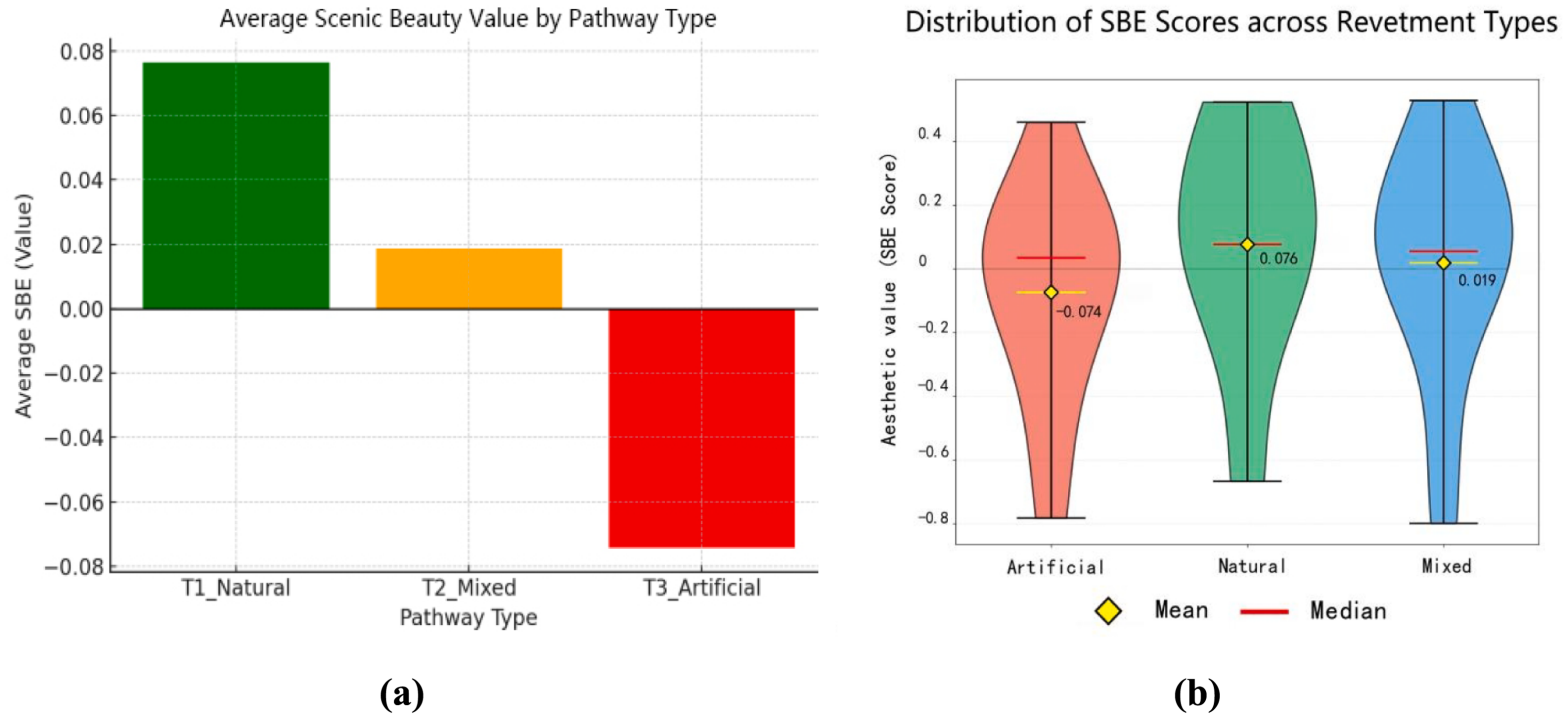
Comparison and distribution patterns of mean SBE scores across different types of waterfront trail landscapes (left: Comparison of mean SBE; right: Distribution patterns of mean SBE; Art: *M* = −0.074, SD = 0.328, *n* = 11; Nat: *M* = 0.076, SD = 0.354, *n* = 8; Mix: *M* = 0.019, SD = 0.353, *n* = 11). **(a)** Average scenic beauty value by pathway type, **(b)** Distribution of SBE scores across revetment types.

Questionnaire results revealed notable variation in respondents’ aesthetic preferences for waterfront trail landscapes (Figure 4). Most participants favored natural elements—such as water bodies, vegetation, and green spaces—as primary contributors to visual appeal and environmental comfort.

#### 3.1.2 Assessing restorative perceptions across different waterfront trail types

The questionnaire results indicated that natural landscapes along waterfront trails had the strongest influence on psychological restoration. Most raespondents agreed that nature-related elements — such as greenery, water bodies, and vegetation — effectively alleviated stress and improved mood. Green and aquatic spaces not only provided visual comfort but also 3x promoted relaxation and emotional regulation, particularly in trails with dense vegetation and abundant water features.

**Figure 5:**
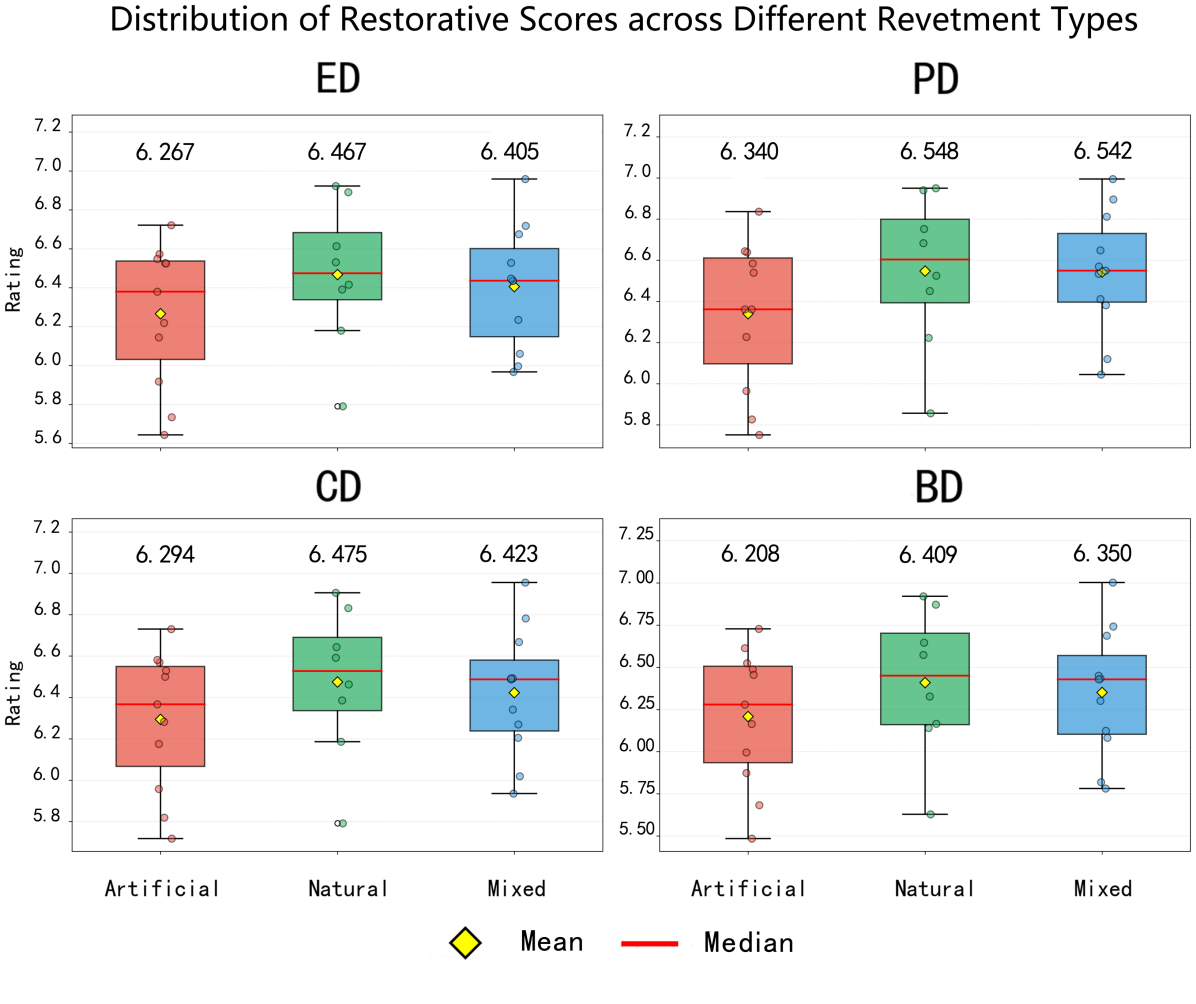
Distribution of restorative scores across different types of waterfront trail landscapes, with mean values indicated.

Restorative analysis across different trail types revealed distinct variations among the three categories in all four dimensions—emotion, cognition, physiology, and behavior (Figure 5).

### 3.2 Relationship between aesthetic preference and restorative perception in waterfront trail landscapes

#### 3.2.1 Relationship between landscape elements and aesthetic preferences across different waterfront trail types

**Figure 6:**
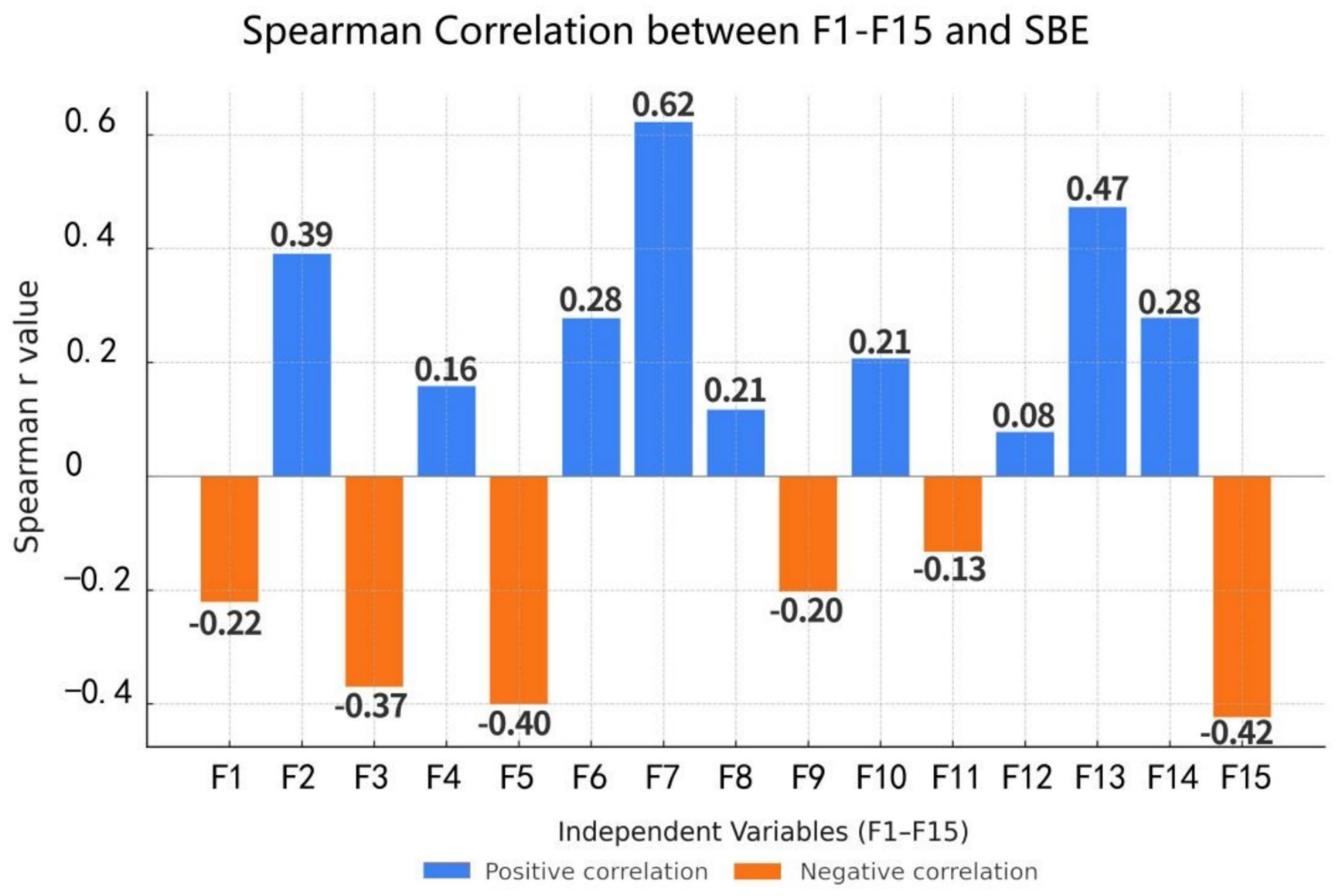
Spearman correlation heatmap illustrating the relationships between 15 landscape variables (F1–F15) and scenic beauty estimation (SBE).

A Spearman correlation analysis was conducted between the 15 landscape variables (F1 – F15) and the SBE values (Figure 6). The results revealed significant positive correlations (*p* < 0.05) between F7 and F13, as well as between F2 and SBE. Conversely, variables such as F15, F5 and F3 showed significant negative correlations with SBE (p < 0.05).

#### 3.2.2 Restorative perceptions across different waterfront trail types

**Figure 7:**
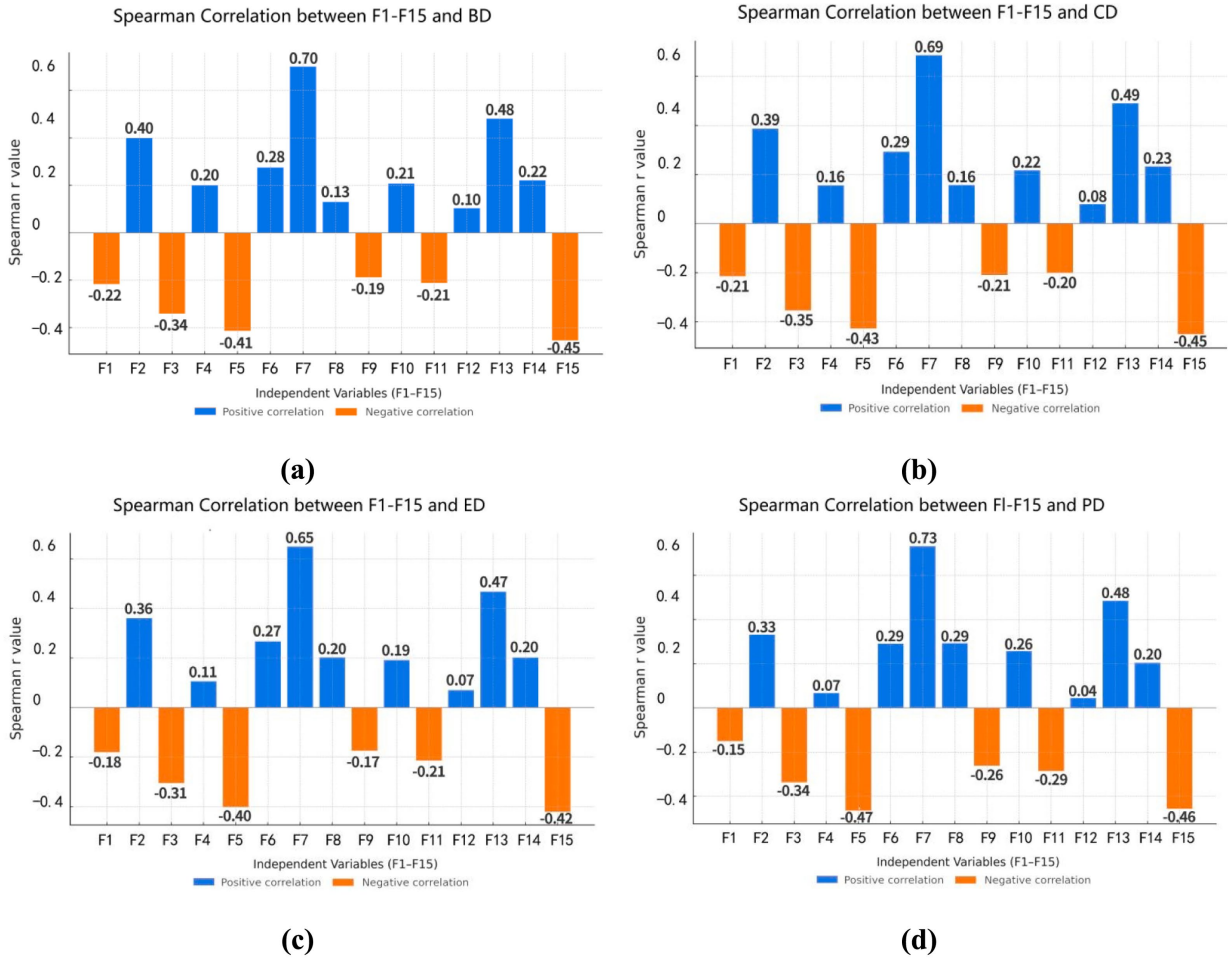
Spearman correlation heatmap illustrating the relationships between 15 landscape variables (F1–F15) and restorative perceptions. **(a)** Spearman correlation between F1-F15 and BD, **(b)** Spearman correlation between F1-F15 and CD, **(c)** Spearman correlation between F1-F15 and ED, **(d)** Spearman correlation between Fl-F15 and PD.

In the BD, F7, F13, and F2 showed significant positive correlations with behavioral scores (p < 0.05). In the CD, the positive associations of F7, F13, and F2 persisted. In the ED, F7 and F13 were positively correlated with emotional restoration. In the PD, F7 and F13 maintained strong positive correlations, while F5 and F15 showed significant negative ones (Figure 7).

### 3.3 Mechanisms linking different waterfront trail types to aesthetic and restorative perceptions

#### 3.3.1 Mechanisms linking landscape elements to aesthetic preferences in waterfront trail

**Figure 8:**
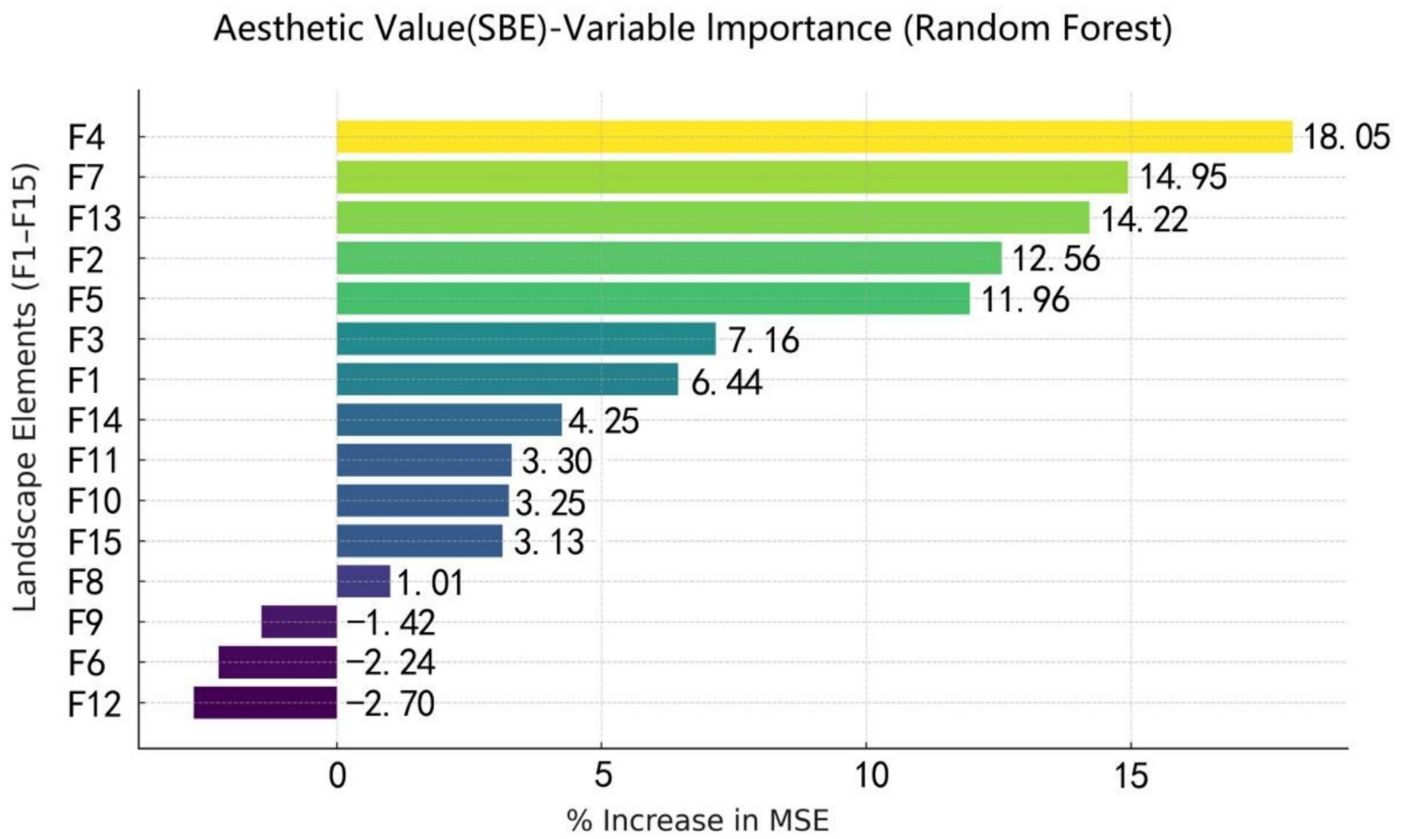
Importance ranking of landscape elements influencing SBE in waterfront trails, derived from the random forest model.

In this study, the Random Forest model was applied to evaluate the relative importance of 15 landscape variables (F1 – F15) in influencing aesthetic preference (Figure 8). The results revealed significant differences in the contributions of individual landscape factors to scenic beauty estimation.

Among all predictors, F4 had the most significant positive impact on aesthetic preference, followed by F7, F13, F2, and F5. In contrast, F9, F6, and F12 exhibited negative effects, with F12 being the strongest.

**Figure 9:**
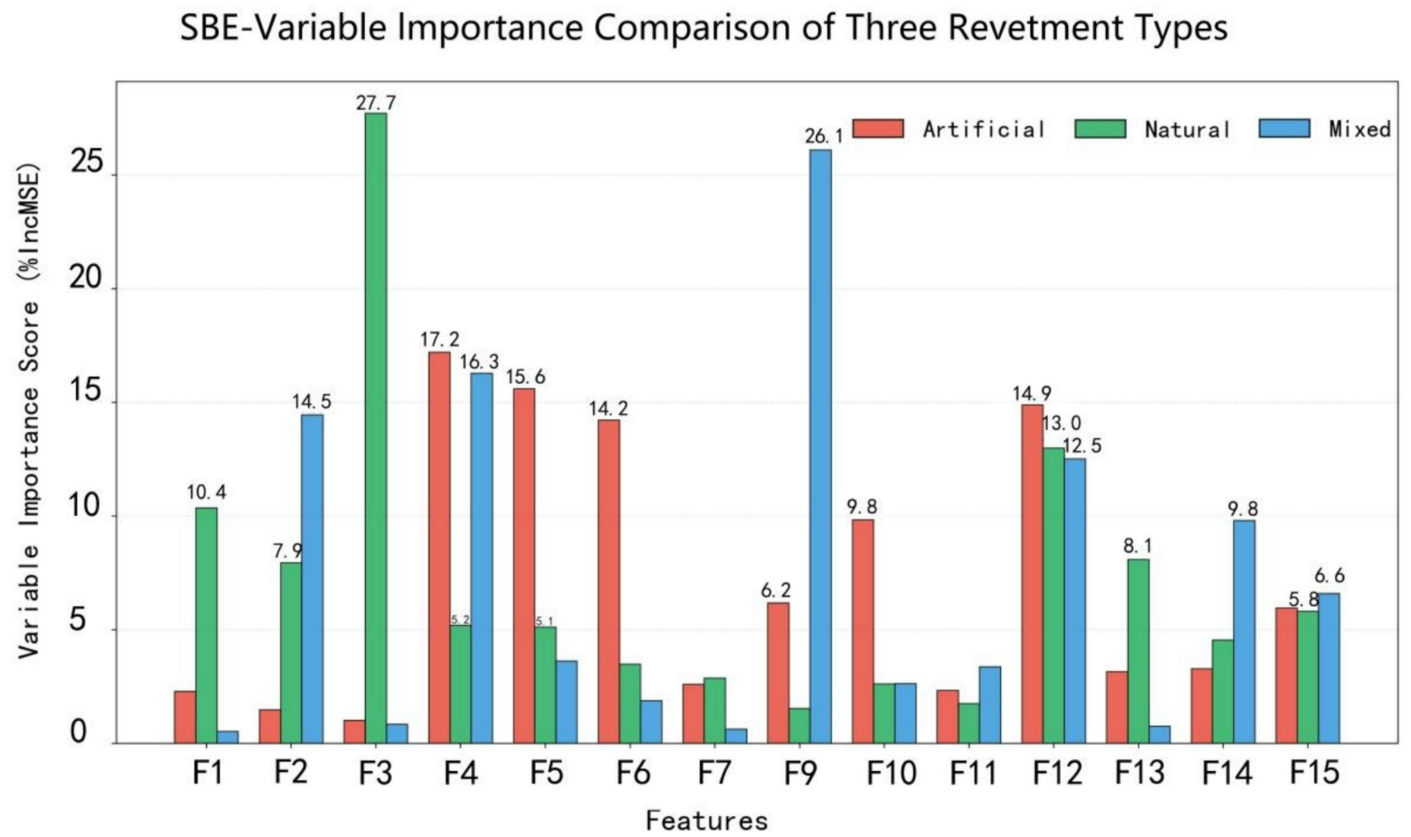
Comparison of feature importance of landscape elements influencing SBE across different waterfront trail types.

Analysis of data from 30 sample sites revealed significant differences in both landscape composition and scenic beauty performance among waterfront trails with different revetment types (Figure 9).

In artificial-type trals (H = 1), F4 exhibited the highest importance, followed by F5 and F12, all scoring higher than the remaining variables.

In natural-type trails (H = 2), the variables displayed distinct ecological characteristics, with F3 showing the highest importance.

In mixed-type trails (H = 3) demonstrated a balanced integration of natural and artificial elements, with F9 being the most influential, followed by F4 and F2.

Across revetment types, the Random Forest results indicate that the natural-type group exhibited the highest predicted SBE, followed by the mixed-type and artificial-type groups. Variables related to natural elements (e.g., F9, F4, and F2) contributed most to SBE prediction in the natural-type group, whereas F4 dominated in the mixed-type group. In the artificial-type group, the relative importance of F5 increased.

#### 3.3.2 Mechanisms linking landscape elements to restorative perceptions in waterfront trail

**Figure 10:**
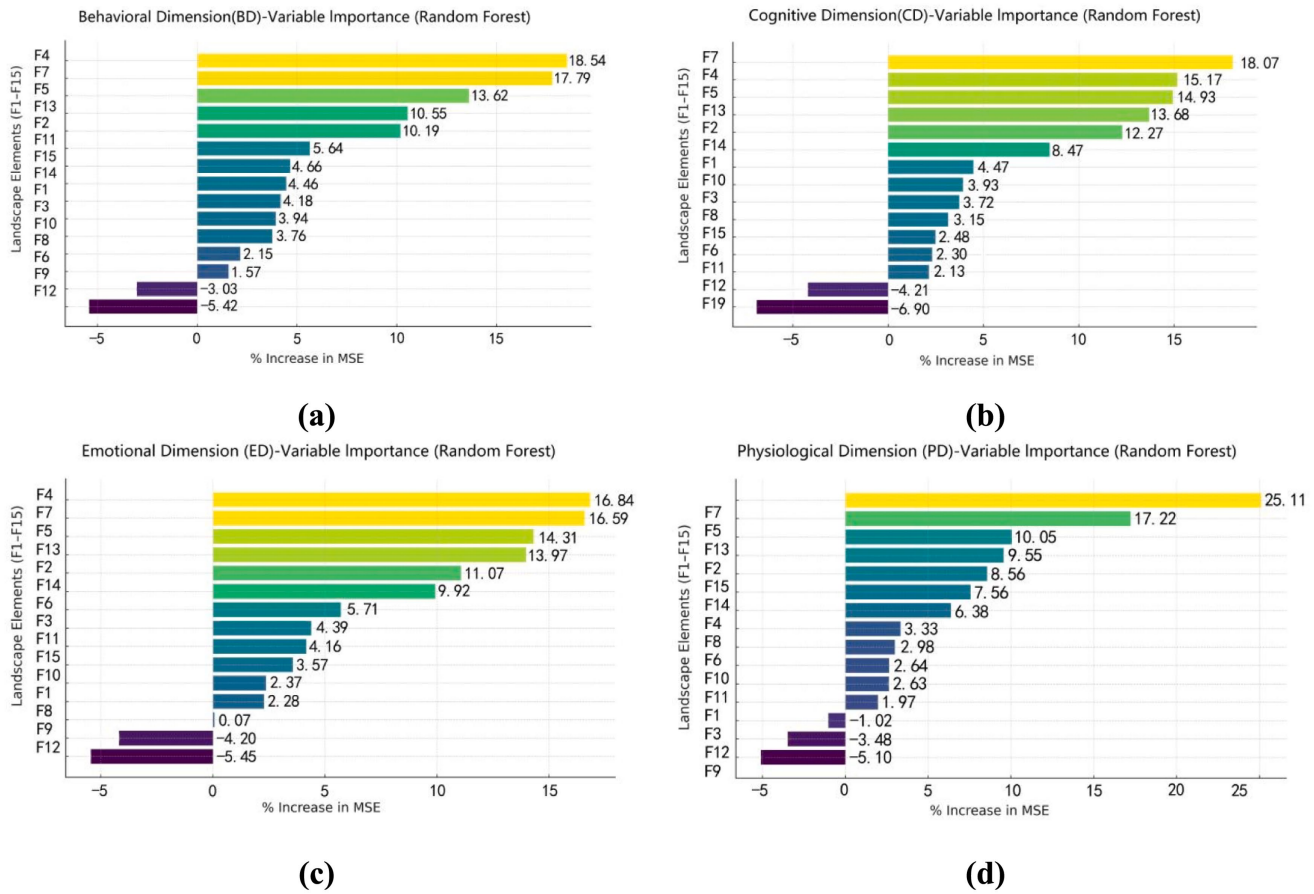
Feature importance analysis of landscape elements influencing different restorative dimensions. **(a)** BD-variable importance based on random forest, **(b)** CD-variable importance based on random forest, **(c)** ED-variable importance based on random forest, **(d)** PD-variable importance based on random forest.

To explore how waterfront trail landscapes influence restorative outcomes, a Random Forest model was used to assess the importance of landscape features across four restorative dimensions (Figure 10).

The BD was primarily driven by F7, F4 and F5. Natural elements including F2 and F13 also contributed positively. In the CD, F7, F4, and F5 were the most influential factors, F13 and F2 also facilitating stable cognitive impressions of the environment. Within the ED, F4, F7 and F5 emerged as the primary triggers of affective responses. Followed by the natural features (F13, F2) increasing feelings of calm and safe. The PD was most strongly associated with F7, followed by F13, F4 and F14.

Across all four dimensions, two factors—F9 and F12—consistently exerted negative effects, indicating that over-artificialization and imbalanced water-area design will undermine both use and restorative experience.

**Figure 11:**
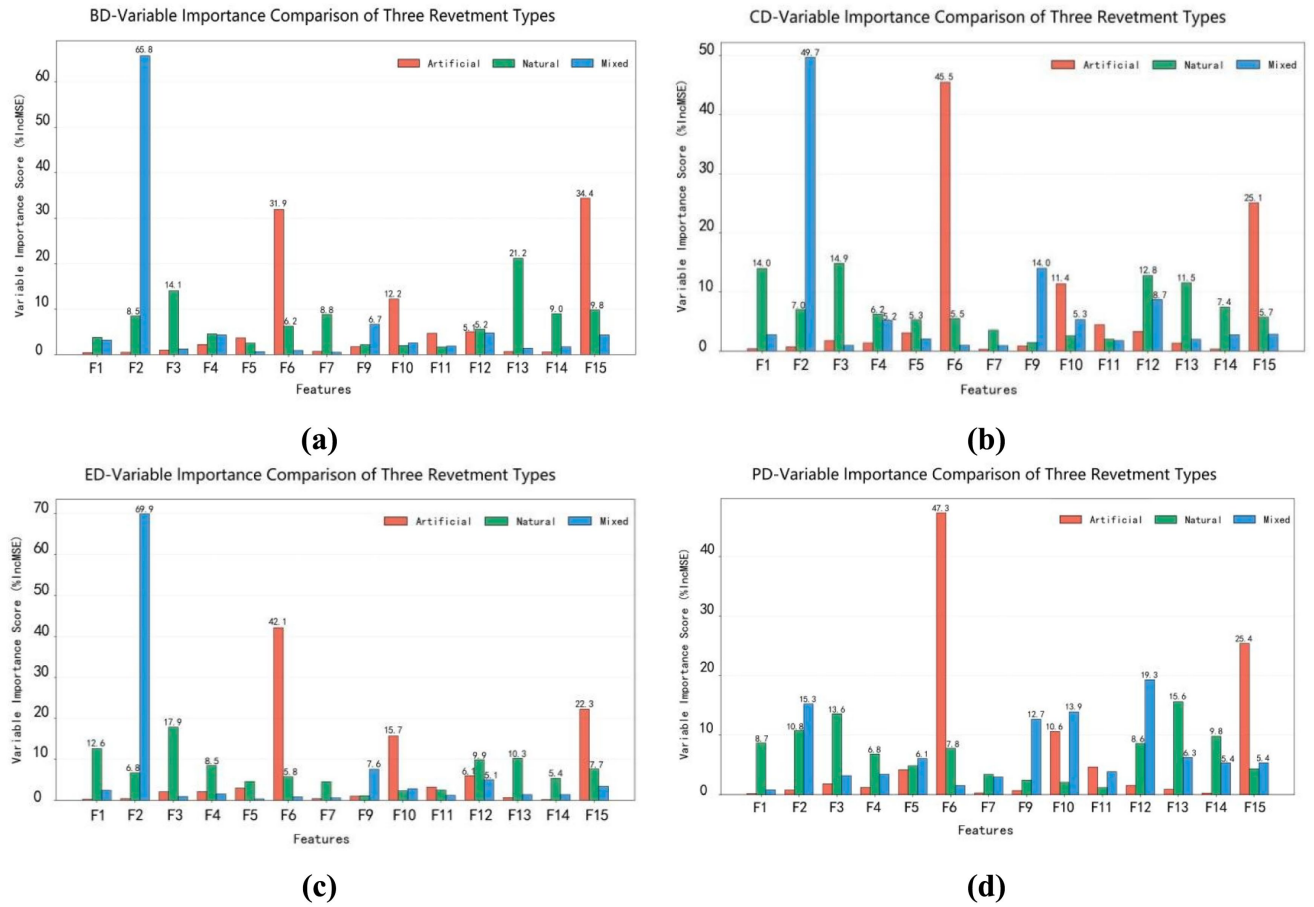
Comparison of the effects of landscape elements on four restorative dimensions across different types of waterfront trails. **(a)** BD-variable importance comparison of three revetment types, **(b)** CD-variable importance comparison of three revetment types, **(c)** ED-variable importance comparison of three revetment types, **(d)** PD-variable importance comparison of three revetment types.

Waterfront trails with different revetment types exhibited distinct patterns across the four restorative dimensions (Figure 11).

In the BD, artificial-type showed higher variable weights for F6 and F15, whereas natural-type were characterized by higher values for F13 and F3. In mixed-type, F2 had the highest score, followed by F9.

In the CD, artificial-type showed greater importance for F6, F15 and F10. In contrast, natural-type were dominated by F3, F1 and F12. For mixed-type, F2 emerged as the most influential factor.

In the ED, artificial-type were primarily influenced by F6, F15 and F10. In natural-type, F3 and F1 showed significant associations, while F2 was again the dominant variable in mixed-type.

In the PD, F6 and F15 had the highest weights in artificial-type, whereas natural-type displayed stronger contributions from F13 and F3. In mixed-type, F12 and F2 were identified as the most important predictors.

### 3.4 Linkages between aesthetic and restorative perceptions across typologically diverse waterfront trails

**Figure 12:**
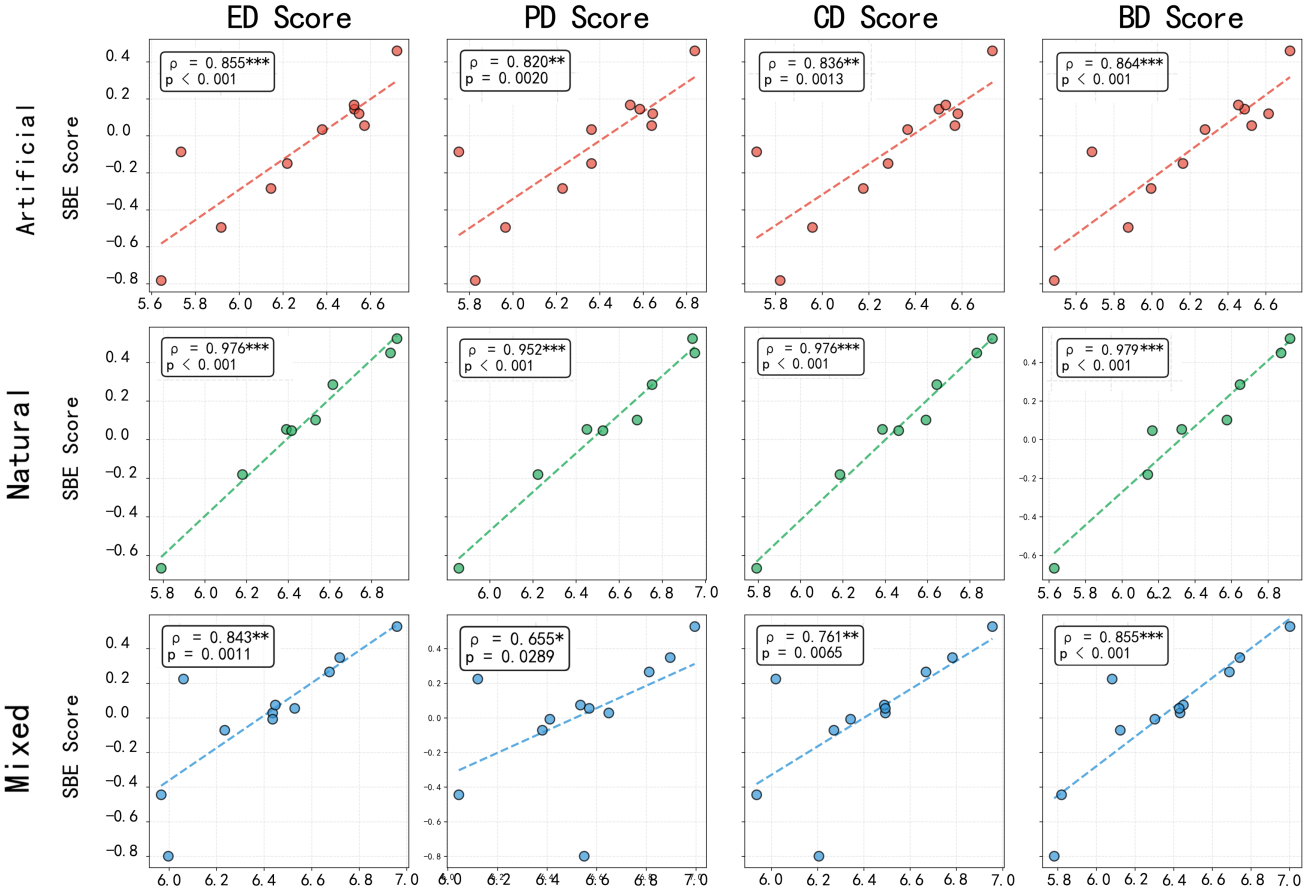
Relationships between SBE and four restorative dimensions (Spearman correlation analysis with scatter plots and regression lines).

As illustrated in Figure 12, distinct correlation patterns were observed between SBE and the four restorative dimensions，across different types of waterfront trails.

For artificial-type trails, SBE exhibited significant positive correlations with all four restorative dimensions (*p* < 0.01), indicating a strong coherence between visual aesthetics and multidimensional restorative experiences. Among these, BD showed the highest correlation with SBE (*ρ* = 0.885, *p* < 0.001), followed by ED (*ρ* = 0.867, *p* < 0.001), while CD (*ρ* = 0.814, *p* = 0.0023) and PD (*ρ* = 0.822, *p* = 0.0019) also demonstrated robust positive associations.

For natural-type trails, SBE was highly correlated with all restorative dimensions, exhibiting near-perfect relationships. ED presented the strongest correlation (*r* = 0.992, *p* < 0.001), closely followed by CD (*r* = 0.991, *p* < 0.001), PD (*r* = 0.986, *p* < 0.05), and BD (*r* = 0.972, *p* < 0.001).

For mixed-type trails, SBE also showed positive correlations with all restorative dimensions. The strongest relationship was observed with BD (*r* = 0.875, *p* < 0.001), followed by ED (*r* = 0.802, *p* = 0.003) and CD (*r* = 0.691, *p* = 0.0186), all of which were statistically significant. Although PD was positively related to SBE (*r* = 0.519, *p* = 0.1016), the correlation was not significant.

**Figure 13:**
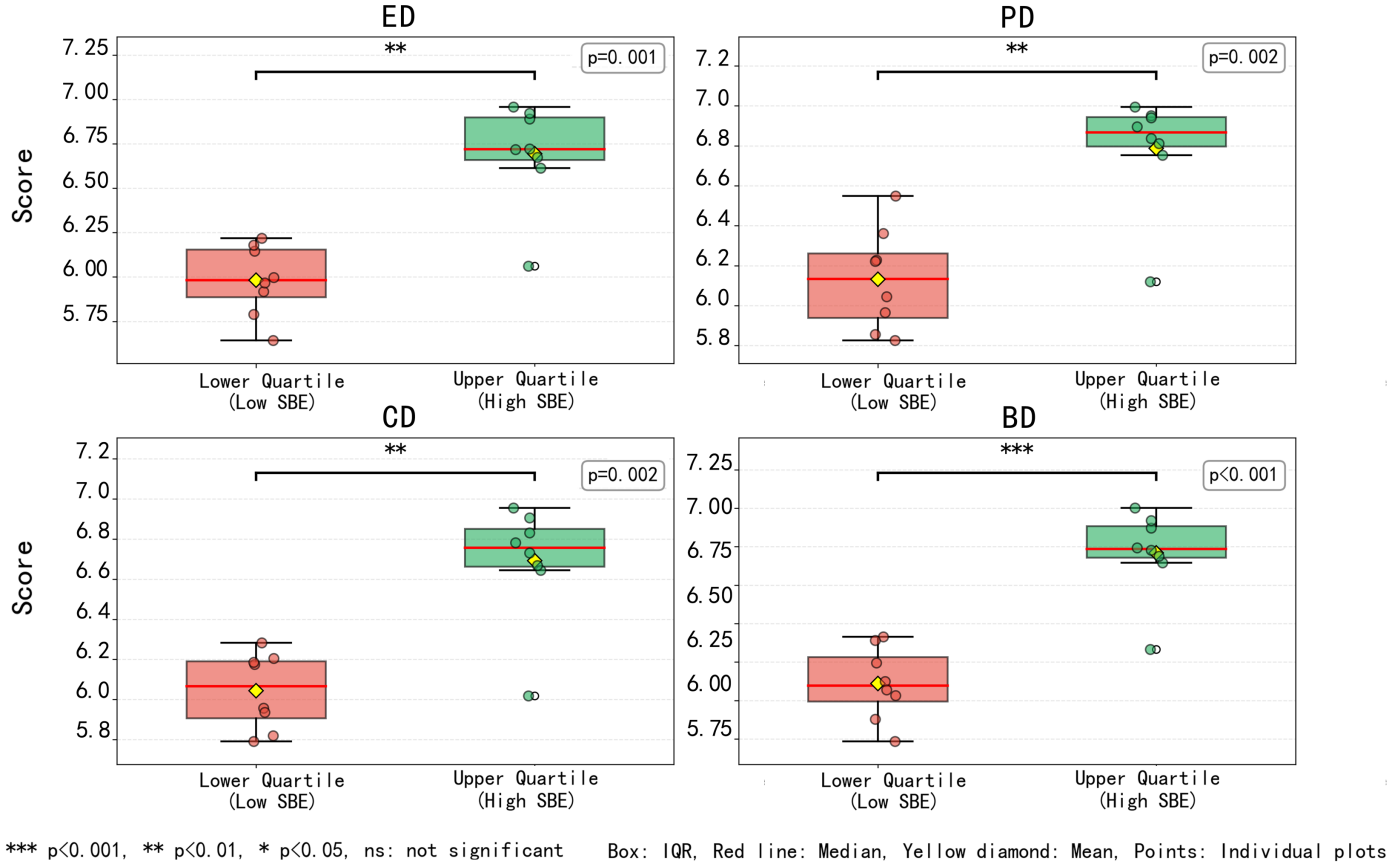
Comparison of scores across four restorative dimensions between upper- and lower-quartile groups.

The upper-quartile sites refer to those ranking at or above the 75th percentile of SBE scores. As shown in Figure 13, sites within the upper quartile exhibited significantly higher scores across all four restorative dimensions compared with lower-quartile sites (all *p* < 0.01). Among these, the differences in the ED and BD dimensions were most pronounced (*p* < 0.001), while significant differences were also observed for the PD and CD dimensions (*p* = 0.002).

To ensure the reliability of statistical comparisons between the two aesthetic preference groups (upper and lower SBE quartiles), four non-parametric methods were jointly applied. Given the relatively small sample size (*n* = 30) and the non-normal distribution of the data, the Mann–Whitney U test was first used to examine median differences between high-aesthetic groups and low-aesthetic groups, providing a robust assessment of significance. A permutation test was then conducted to validate these results through repeated resampling, minimizing potential bias from distributional assumptions. Non-parametric comparisons between high- and low-SBE groups showed significant differences across all restorative dimensions with large effect sizes (Table 4); bootstrap-based confidence intervals confirmed the robustness of these differences.

**Table 4:** Non-parametric test results comparing four restorative dimensions between upper and lower-quartile groups.

Dimension	Lower Quartile (*n* = 8)	Upper Quartile (*n* = 8)	U	*p*-value	Effect Size (r)	Cliff ‘s δ	95% CI
ED	5.98 (0.27)	6.72 (0.24)	3.0	0.001**	0.761	0.906	[0.51, 0.99]
PD	6.13 (0.32)	6.87 (0.15)	4.0	0.002**	0.735	0.875	[0.48, 1.01]
CD	6.07 (0.28)	6.76 (0.19)	4.0	0.002**	0.735	0.875	[0.47, 0.95]
BD	5.85 (0.29)	6.74 (0.21)	2.0	<0.001***	0.788	0.938	[0.59, 1.13]

The combined application of these four approaches enhanced the robustness and credibility of the statistical inferences. The Mann–Whitney U test offered the primary test of significance. Overall, all four restorative dimensions showed statistically significant differences between the high-SBE groups and low-SBE groups, with large effect sizes across all metrics, indicating that the statistical analyses were both reliable and highly explanatory (Table 4).

### 3.5 Structural equation modeling results

**Figure 14:**
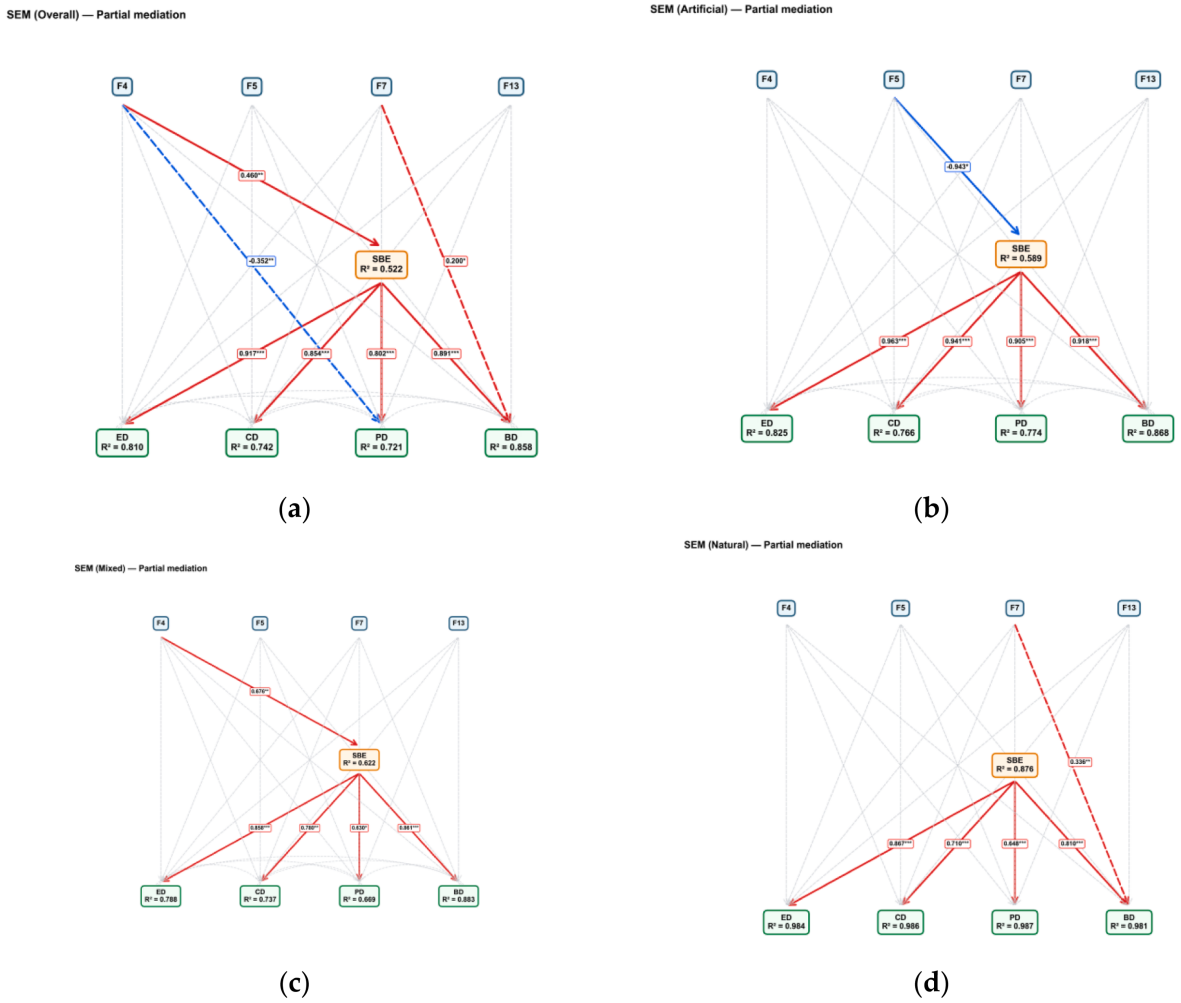
SEM path model results for the overall sample and different types of revetments solid: Mediated paths (significant only), dashed: Direct paths (sig labeled; non-sig gray, unlabeled). ** p < 0.001, * p < 0.01, *p < 0.05. **(a)** SEM with overall-partial mediation, **(b)** SEM with artificial-partial mediation, **(c)** SEM with mixed-partial mediation, **(d)** SEM with natural-partial mediation.

In the partial mediation model for the overall sample (Overall; Figure 14a), the explanatory contribution of landscape factors to SBE is primarily derived from F4, as evidenced by a significant positive effect of F4 on SBE (*β* = 0.460). SBE, in turn, exerts significant positive effects on all four restorative dimensions, namely ED (*β* = 0.917), CD (*β* = 0.854), PD (*β* = 0.802), and BD (*β* = 0.891), indicating that SBE plays a key mediating role between landscape factors and restorative dimensions. In addition to the mediated pathways, the overall model also identifies two significant direct effects: a significant negative path from F4 to PD (*β* = −0.352) and a significant positive path from F7 to BD (*β* = 0.200). These results suggest that some landscape effects are not fully transmitted through SBE, consistent with the characteristics of a partial mediation model. The model explains 52.2% of the variance in SBE (*R*^2^ = 0.522), while the explained variances for ED, CD, PD, and BD are 0.810, 0.742, 0.721, and 0.858, respectively. Other non-significant direct paths are indicated by gray dashed lines without coefficient labels. Full results for model fit indices and key path coefficients are provided in Supplementary Tables S1–S5.

Grouped analyses further reveal differences in path structures across different revetment types (Figures 14b–d). In artificial revetments, only the path from F5 to SBE is significant and negative (*β* = −0.943), whereas the paths from SBE to ED, CD, PD, and BD are all significantly positive with relatively high coefficients (*β* = 0.905–0.964). Correspondingly, *R*^2^(SBE) is 0.589, and the R^2^ values for ED, CD, PD, and BD are 0.825, 0.766, 0.774, and 0.868, respectively. In mixed revetments, the dominant landscape-driven path shifts to a significant positive effect of F4 on SBE (*β* = 0.676). SBE continues to significantly and positively predict all four dimensions (ED: *β* = 0.858; CD: *β* = 0.780; PD: *β* = 0.630; BD: *β* = 0.861), with *R*^2^(SBE) = 0.622 and R^2^ values of 0.788, 0.737, 0.669, and 0.883 for ED, CD, PD, and BD, respectively. In natural revetments, SBE maintains significant positive effects on all four dimensions (*β* = 0.648–0.867), while a significant positive direct effect of F7 on BD emerges (*β* = 0.336), indicating a more pronounced partial mediation pathway for the BD dimension. This group exhibits the highest explanatory power, with *R*^2^(SBE) = 0.876 and *R*^2^ values of 0.984, 0.986, 0.987, and 0.981 for ED, CD, PD, and BD, respectively.

Detailed model fit indices and full standardized path coefficients are reported in the Supplementary materials.

## 4 Discussion

### 4.1 Tourists’ aesthetic preferences toward landscape elements in different types of waterfront trails

The integrated results of this study identified F4, F7, and F13 as the key landscape factors jointly influencing SBE and the psychological restoration of waterfront trails. Among them, F4 and F7 showed the highest overall importance, exerting strong effects on both aesthetic preference and restorative experience. The integration of architectural and green elements further enhanced perceived attractiveness and environmental comfort. These findings are consistent with those of Xiaojia et al. (36).

Feature importance analysis based on the Random Forest model corroborated these results, confirming that F4, F7, and F13 contributed most significantly to predicting aesthetic preference. Prior studies have likewise highlighted that the visual characteristics of pavement surfaces—such as color, pattern, and texture—and their harmony with the surrounding environment substantially affect aesthetic judgments (37). Moreover, coherent spatial organization and balanced integration of natural components were found to strengthen ecological perception and visual comfort, thereby promoting psychological restoration. These results align with Ekkel et al. (38), who identified the positive perceptual impacts of green-space accessibility, and Zohre et al. (39), who demonstrated a strong positive association between green view rate and scenic beauty.

Different types of waterfront trails exhibited distinct aesthetic preference patterns, shaped by the varying influence of multiple landscape elements. In natural-type trails, F3 showed the highest explanatory power, followed by F12 and F1, indicating that spatial enclosure and the degree of hydrological enclosure are decisive factors in enhancing visual pleasure and supporting psychological restoration. In artificial-type trails, F4, F5, and F12 emerged as the dominant contributors, suggesting that chromatic diversity, the proportion of built forms, and waterfront articulation jointly define the visual appeal and perceived aesthetic quality of constructed landscapes. For mixed-type trails, F9 and F4 displayed the highest importance, highlighting that, in environments where natural and artificial elements coexist, the coordination between service-oriented facilities and color composition plays a primary role in shaping overall aesthetic perception. These findings are consistent with Hofmann et al. (40), who identified systematic perceptual variations among urban green-space typologies and emphasized that environmental structure fundamentally governs the sources of landscape attractiveness. Overall, natural-dominant environments tend to yield higher restorative and aesthetic benefits, while artificial-dominant spaces depend more on design articulation and spatial coherence to achieve aesthetic quality.

However, certain discrepancies were observed when compared with previous research. Specifically, this study found that F15 and F3 exhibited weak or even negative relationships with aesthetic preference, whereas some studies have suggested that moderate visual complexity can enhance landscape attractiveness and perceived naturalness (41). This inconsistency likely arises from contextual differences: the present study focuses on highly urbanized waterfront trails, where exposed or visually cluttered areas are often interpreted as lacking cleanliness or safety, thus reducing overall scenic appeal. In contrast, research conducted in natural reserves or suburban parks tends to value ecological complexity and visual heterogeneity as indicators of authenticity and naturalness rather than disorder.

### 4.2 Effects of waterfront trail typologies on restorative perceptions

Regarding restorative outcomes, F2, F7 and F13 exerted consistently positive and significant effects across the four restorative dimensions. Pavement form enhanced walking comfort and physiological recovery through its smooth, natural texture, while its continuous visual pattern contributed to psychological relaxation and a sense of stability. These findings are consistent with the principles of evolutionary psychology (42, 43), which emphasize that organized and legible environments foster emotional calm and adaptive behavior.

Within the BD, F4 and F7 were identified as the strongest predictors of engagement and stay behaviors. Diverse yet harmonious color compositions enhanced visual appeal and enjoyment, stimulating emotional activation and encouraging outdoor participation, whereas smooth and coherent paving directly improved walking comfort and perceived safety. Previous studies have emphasized that environmental safety and aesthetic appeal are key determinants of outdoor activity levels (44, 45). In addition, F13 and F2 showed moderate positive associations, suggesting that spatial enclosure and visibility of natural features interact synergistically to facilitate behavioral restoration. Moderate enclosure enhances feelings of safety and orientation, while a higher proportion of visible greenery strengthens ecological ambiance and spatial attractiveness. However, excessive vegetation density or overly confined spaces may reduce openness and perceived accessibility, thereby suppressing behavioral recovery. Shibata et al. (46) further confirmed that variations in greenery density significantly affect visual perception and restorative experience.

In the CD, F7, F4, and F5 were identified as the primary factors influencing restorative experience. A well-designed paving system provides a smooth and continuous walking surface, reducing cognitive load and improving spatial legibility. Variations in color enhance visual recognition and aesthetic pleasure, while the moderate presence of architectural structures introduces spatial guidance and order, thereby facilitating cognitive restoration. These findings are consistent with those of Zhang et al. (47) and Chiang et al. (48), who emphasized that paving continuity and spatial legibility enhance cognitive mapping and attentional restoration. Similarly, Bell et al. (49) demonstrated that visual complexity and color variation facilitate attention recovery by stimulating moderate visual engagement without inducing overload.

In the ED, F4, F7 and F5 exerted the greatest influence. Harmonious and warm color tones were found to significantly improve emotional states, whereas comfortable paving and appropriate architectural enclosure jointly shaped a sense of safety and pleasantness. These elements enhanced the overall environmental affinity and contributed to emotional recovery within the waterfront space. These results align with those of Zhang et al. (50), whose empirical and review studies indicated that warm color tones and coherent paving textures enhance perceived safety, pleasure, and emotional recovery. Likewise, Gulwadi et al. (51) found that spatial enclosure and architectural harmony in campus landscapes evoke a stronger sense of place and emotional well-being.

In the PD, F7 and F5 were the most influential variables. Continuous and even paving surfaces improved walking comfort and reduced physical fatigue, while appropriately scaled architectural features helped regulate microclimatic conditions and visual pressure. Together, these factors contributed to physical relaxation and facilitated physiological restoration. Some studies, however, suggest that moderate levels of built facilities can enhance convenience and functional support (52). The divergence likely results from variations in facility density and design quality: overly dense or poorly integrated structures may interfere with natural perception and weaken restorative effects.

Distinct restorative mechanisms were also evident among the three trail typologies. Natural-type trails exhibited the strongest overall restorative performance across all four dimensions, particularly within the ED and CD domains. High green-view ratios and diverse vegetation compositions promoted positive affect and reduced physiological stress, in accordance with Attention Restoration Theory (ART), which posits that natural environments restore cognitive capacity through involuntary attention (49). Artificial-type trails also showed significant positive correlations across all restorative dimensions, with BD and ED presenting the highest associations. Feature importance analysis revealed that restorative potential in artificial trails was primarily driven by F7, F15 and F5. Pavement form consistently functioned as the core determinant across all dimensions, suggesting that coherent, even paving not only enhances physical comfort but also strengthens spatial order and psychological stability. Overall, the restorative effects of artificial trails appear to rely on visual order, structural clarity, and functional controllability—supporting findings from urban psychology that legibility and perceived safety are key facilitators of mental recovery (49). Mixed-type trails, combining both natural and artificial elements, exhibited multidimensional restorative synergy. Correlation analysis indicated that SBE was most strongly associated with BD and ED, followed by CD, while PD showed a weaker, non-significant relationship. This pattern suggests that mixed-type trails more effectively promote emotional pleasure and behavioral engagement, though their influence on physiological relaxation remains limited. Feature importance analysis further demonstrated that F2 played a dominant role across multiple dimensions, particularly in ED and CD, indicating that variability in spatial scale and enclosure substantially shapes psychological recovery and cognitive focus. These findings are consistent with those of Yan et al. (53), who reported that differences in the spatial scale of urban green spaces significantly contribute to improvements in psychological well-being.

### 4.3 Interrelations and theoretical foundations linking aesthetic preference and restorative experience

This study identified a stable and significant positive relationship between aesthetic preference and restorative perception. Sites with higher aesthetic evaluation scores achieved consistently higher ratings across all four restorative dimensions, underscoring the close association between visual attractiveness and multidimensional restoration. This finding aligns with the systematic conclusions of Van Valkengoed Anne et al. (54). Analysis of the emotion and physiology data further revealed that sites in the upper quartile of aesthetic preference scored significantly higher in both the ED and PD. This indicates that highly aesthetic environments effectively mitigate negative emotions and psychological stress while promoting physical relaxation. Similarly, Van den Berg et al. (55) demonstrated that aesthetic preference across natural environments is strongly correlated with stress alleviation, lending support to the physiological mechanisms observed in this study. The CD also showed a clear upward trend, suggesting that visually attractive environments can reduce cognitive load and enhance attention and mental stability. In the BD, high-aesthetic sites encouraged greater user engagement and participation, with respondents more inclined to linger, explore, or engage in social interactions. Collectively, these patterns highlight the pivotal role of visual features in facilitating restorative experiences along waterfront trails.

The Random Forest analysis further reinforced this relationship. F2, F7, F13 and F12 emerged as the most influential predictors in both the aesthetic preference and restorative perception models. This convergence indicates that visually appealing landscape elements also tend to offer strong functional support. Among natural features, F13 made the greatest contribution—a finding validated across diverse environmental contexts by Shibata et al. (46). Moreover, the sense of safety and visual orientation provided by F2 corresponds with the findings of Van den Berg et al. (55), who emphasized the spatial attributes of highly restorative environments.

Aesthetic preference indirectly fosters emotional regulation, cognitive relaxation, and behavioral engagement by evoking positive emotions and visual pleasure. The F13 enhances visual quality, alleviates psychological stress, and promotes emotional stability through diverse vegetation layering. F2 operates on both perceptual and psychological levels: moderate enclosure minimizes external disturbance and enhances the sense of security, whereas excessive confinement reduces visual comfort. F11 and F9 played a limited but supportive role in restoration, primarily improving safety and behavioral convenience through shoreline design and amenity accessibility. Conversely, F5 and F15 showed negative associations with both aesthetic preference and restoration, as their prevalence disrupts natural ambiance and visual coherence, diminishing environmental appeal and psychological comfort. While some studies suggest that moderate built structures can enhance convenience (52), excessive density or imbalance between artificial and natural elements clearly weakens restorative effects. Importantly, the underlying mechanisms differ: restorative experience is more responsive to spatial organization and functional configuration, whereas aesthetic experience is primarily driven by visual form and compositional harmony. For example, F11 and F9 had greater importance in restorative dimensions than in aesthetic preference, indicating that safety and usability are more critical to physical and psychological recovery. In contrast, F5 and F15, though potentially adding visual diversity, did not effectively facilitate mental relaxation. Overall, the aesthetic and restorative functions of landscapes are not fully congruent; they operate through distinct pathways—the former emphasizing “visual appeal and beauty,” and the latter relying more on “supportiveness and comfort.” This distinction underscores the need for waterfront trail design to balance visual expression with restorative functionality. Luo et al. (56) similarly emphasized that identifying and strengthening shared landscape features that simultaneously enhance aesthetic and restorative outcomes represents a key direction for effective landscape intervention.

Overall, a significant and coherent positive relationship exists between aesthetic preference and restoration in waterfront trails. Landscape elements of high aesthetic value often provide substantial functional support, contributing to emotion, cognition, behavior, and physiology recovery. These findings offer practical guidance for the evidence-based design of waterfront trails, highlighting that the pursuit of visual aesthetics should be accompanied by the coordination of spatial structure and natural elements to achieve dual optimization of aesthetic appeal and restorative potential.

### 4.4 SEM-based mechanistic interpretation and typological differences

The SEM results corroborate previous analyses. In the overall sample, SBE shows consistently positive associations with ED, CD, PD, and BD, confirming that aesthetic preference serves as a central pathway through which pathway landscapes enhance multidimensional restorative experiences. However, the presence of several significant direct effects indicates that not all landscape influences are fully mediated by SBE. This suggests a dual-mechanism structure of restoration, involving both an indirect pathway driven by aesthetic evaluation and a more direct pathway reflecting immediate environmental responses to specific landscape attributes.

Path structures further diverge across revetment types. In artificial revetments, SBE is primarily associated with F5, highlighting the dominant role of built-element representation in shaping aesthetic judgments. In mixed revetments, the stronger linkage between F4 and SBE underscores the increased importance of visually expressive features in transitional landscape settings. In natural revetments, alongside the stable mediating role of SBE, a direct effect of F7 on BD emerges, suggesting that under more natural conditions, certain spatial or material characteristics may directly facilitate behavioral or bodily restoration. Together, these findings demonstrate that the restorative mechanisms of waterfront pathways are context-dependent, with landscape type modulating the relative contributions of aesthetic mediation and direct environmental effects.

### 4.5 Limitations and prospects

This study has certain limitations, primarily related to the insufficient consideration of multisensory environmental factors. Zhang et al. (57) reported in a study conducted in Guangzhou that, in addition to visual elements, other sensory dimensions such as auditory and tactile factors also have significant effects on restorative experiences. This differs from the visual-centric analytical framework adopted in the present study. Although this research provided an in-depth exploration of visual characteristics and their restorative effects, it did not adequately account for non-visual components such as soundscape and thermal comfort, which may partly limit the interpretation of the physiological restoration mechanisms (52). Future research could integrate multisensory monitoring and evaluation methods—combining soundscape analysis, microclimate data collection, and synchronized recording of physiological responses—to more comprehensively elucidate the combined influence of multisensory environments on psychological and physiological restoration. Such an approach would address the limitations of a purely visual analysis and enhance both the applicability and explanatory depth of the findings.

In addition, there is a limitation, this study is the relatively small effective sample size (*n* = 30 scenes) in relation to the number of variables considered and the analytical complexity of the modeling framework. Although multiple advanced analytical techniques were employed, such a sample size may increase the risk of overfitting and model instability, the stability of model estimates and feature rankings may be sensitive to data perturbations，and may limit the generalizability of the results beyond the studied dataset. To mitigate these concerns, the analyses were designed with an exploratory objective, focusing on identifying robust patterns and consistent relationships rather than generating definitive or highly parameterized models. Future research should prioritize larger and more diverse datasets, to improve model stability, reduce overfitting risk, and more robustly assess the generalizability of the identified relationships.

## 5 Conclusion

This study integrated image semantic segmentation and Random Forest modeling to quantitatively assess the landscape characteristics of waterfront trails in Fuzhou, examining how different landscape elements shape users’ perceptual experiences in terms of aesthetic preference and psychological restoration. The results demonstrated that aesthetic quality and restorative experience exhibited a parallel enhancement pattern, suggesting that the quality of landscape design plays a critical role in shaping the public’s sense of pleasure and psychological well-being. Distinct performance patterns were observed across trail types: natural-type trails, characterized by vegetation and water features, exerted the strongest effects on emotion and physiology restoration; artificial-type trails depended primarily on the optimization of paving materials and built facilities; whereas mixed-type trails achieved a more balanced performance through the coordinated integration of natural and artificial components. Higher levels of vegetation coverage, well-proportioned spatial enclosures, and the use of natural, comfortable paving materials significantly enhanced both aesthetic appeal and psychological relaxation. Theoretically, the findings deepen the understanding of the interplay between aesthetic perception and restorative experience in waterfront landscapes. Practically, they provide a scientific foundation and methodological framework for the planning and design of urban waterfront trails and blue–green infrastructure. Future waterfront development should place greater emphasis on the integration of natural elements and the balance of spatial comfort. Enhancing visual greenery, optimizing trail configurations, and establishing multi-layered landscape environments can collectively foster ecological aesthetics and human well-being, thereby achieving a synergistic enhancement of environmental quality and restorative potential.

## Data Availability

The original contributions presented in the study are included in the article/Supplementary material, further inquiries can be directed to the corresponding author.
