# Supplementary material for: Assessing the impact of waterfront trail aesthetics on psychological restoration in urban environments: a deep learning and random forest approach
Source: Front Public Health. 2026 Jan 30;13:1757145. doi: 10.3389/fpubh.2025.1757145 (PMC12901393; doi:10.3389/fpubh.2025.1757145)
Supplement: Supplementary file 1 [file Table_1.docx]

**Table 1.** Classification of Spatial Types and Site Selection of waterfront trails in Fuzhou

| **Number** | **Region** | **Sample Point** | **Structural Type** | **Number** | **Region** | **Sample Point** | **Structural Type** |
| --- | --- | --- | --- | --- | --- | --- | --- |
| 1 | Jin'an  District | JA-ZSSL | Artificial  Revetment | 16 | Gulou  District | GL-BM-5 | Mixed  Revetment |
| 2 | Taijiang  District | TJ-GMG-1 | Natural  Revetment | 17 |  | GL-BM-6 | Natural  Revetment |
| 3 |  | TJ-NGY-1 | Mixed  Revetment | 18 |  | GL-BM-7 | Natural  Revetment |
| 4 | Cangshan  District | CS-AMS | Natural  Revetment | 19 |  | GL-BM-9 | Artificial  Revetment |
| 5 |  | CS-BY-2 | Artificial  Revetment | 20 |  | GL-DX-3 | Mixed  Revetment |
| 6 |  | CS-DL-2 | Artificial  Revetment | 21 |  | GL-DX-4 | Artificial  Revetment |
| 7 |  | CS-JH | Artificial  Revetment | 22 |  | GL-DX-5 | Artificial  Revetment |
| 8 |  | CS-HXN-3 | Mixed  Revetment | 23 |  | GL-FM-2 | Artificial  Revetment |
| 9 |  | CS-LP-2 | Artificial  Revetment | 24 |  | GL-GY-1 | Artificial  Revetment |
| 10 |  | CS-HWB | Mixed  Revetment | 25 |  | GL-HP-2 | Artificial  Revetment |
| 11 |  | CS-YQ-1 | Natural  Revetment | 26 |  | GL-HQ-1 | Artificial  Revetment |
| 12 |  | CS-ZY | Mixed  Revetment | 27 |  | GL-JA-3 | Mixed  Revetment |
| 13 | Gulou  District | GL-BM-1 | Natural  Revetment | 28 |  | GL-WS-1 | Artificial  Revetment |
| 14 |  | GL-BM-2 | Natural  Revetment | 29 |  | GL-XX-2 | Mixed  Revetment |
| 15 |  | GL-BM-3 | Natural  Revetment | 30 |  | GL-XX-6 | Artificial  Revetment |

**Table 2.** SRRS Scale Dimensions and Items

| **Dimensions** | **Item** | **Serial number** |
| --- | --- | --- |
| Emotion | How would you describe the impact of scenery on your emotional changes in the following environment? |  |
|  | Depressed → Happy | A1 |
|  | Anxious → Relaxing | A2 |
|  | Exhausted → Full of vitality | A3 |
| Physiology | How would you describe the physiological response caused by the scenery in the following environment? |  |
|  | My breathing is accelerating. | B1 |
| Cognition | How would you describe the impact of landscape on your cognition in the following environment? |  |
|  | I am very interested in the current environment. | C1 |
|  | My mental exhaustion is decreasing. | C2 |
| Behavior | Which behavior would you identify with in the following environment? |  |
|  | I want to explore this place more deeply. | D1 |
|  | I want to visit here more often. | D2 |

**Table 3.** Landscape Element Decomposition Table of Urban Waterfront Trail

| **Type** | **Landscape Elements** | **Computational Method** | **Quantitative Method** | **Serial number** |
| --- | --- | --- | --- | --- |
| Spatial  Elements | Sky Openness | The proportion of the sky in the landscape view | Image Semantic Segmentation | F1 |
|  | Spatial Enclosure | The proportion of vertical elements in the landscape view | Image Semantic Segmentation | F2 |
|  | Visual Complexity | Complex index of landscape space composition | Matlab | F3 |
|  | Color Richness | Color richness index of landscape spatial elements | Matlab | F4 |
| Artificial Elements | Building Elements | The proportion of urban buildings in the landscape view | Image Semantic Segmentation | F5 |
|  | Pavement Elements | The proportion of hard pavement in the landscape view | Image Semantic Segmentation | F6 |
|  | Pavement Pattern | Cement = 1, Asphalt = 2,  Bricks and Stones = 3, Wood = 4 | Subjective Assignment | F7 |
|  | Revetment Type | Artificial =1 ,Natural = 2,  Artificial + Natural = 3 | Subjective Assignment | F8 |
|  | Facility Elements | The proportion of landscape service facilities in the landscape view | Image Semantic Segmentation | F9 |
|  | Small Structural Elements | The proportion of decorative small structures in the landscape view | Image Semantic Segmentation | F10 |
|  | Waterfront Enclosure Degree | Degree of water boundary enclosure | Image Semantic Segmentation | F11 |
| Natural  Elements | Blue View Rate | The proportion of water bodies in the landscape view | Image Semantic Segmentation | F12 |
|  | Green View Rate | The proportion of vegetation in the landscape view | Image Semantic Segmentation | F13 |
|  | Vegetation Layers | The proportion of plants of different heights in the landscape view | Image Semantic Segmentation | F14 |
|  | Soil Exposure Degree | The proportion of bare soil in the field of view | Image Semantic Segmentation | F15 |

**Table 4.** Non-parametric test results comparing four restorative dimensions between upper- and lower-quartile groups

| **Dimension** | **Lower Quartile (n** **=** **8)** | **Upper Quartile (n** **=** **8)** | **U** | **p-value** | **Effect Size (r)** | **Cliff** **'s δ** | **95% CI** |
| --- | --- | --- | --- | --- | --- | --- | --- |
| ED | 5.98 (0.27) | 6.72 (0.24) | 3.0 | 0.001** | 0.761 | 0.906 | [0.51, 0.99] |
| PD | 6.13 (0.32) | 6.87 (0.15) | 4.0 | 0.002** | 0.735 | 0.875 | [0.48, 1.01] |
| CD | 6.07 (0.28) | 6.76 (0.19) | 4.0 | 0.002** | 0.735 | 0.875 | [0.47, 0.95] |
| BD | 5.85 (0.29) | 6.74 (0.21) | 2.0 | <0.001*** | 0.788 | 0.938 | [0.59, 1.13] |
